# ULK1-mediated phosphorylation regulates the conserved role of YKT6 in autophagy

**DOI:** 10.1242/jcs.260546

**Published:** 2023-02-10

**Authors:** Pablo Sánchez-Martín, Franziska Kriegenburg, Ludovico Alves, Julius Adam, Jana Elsaesser, Riccardo Babic, Hector Mancilla, Mariya Licheva, Georg Tascher, Christian Münch, Stefan Eimer, Claudine Kraft

**Affiliations:** ^1^ Institute of Biochemistry and Molecular Biology, ZBMZ, Faculty of Medicine, University of Freiburg, 79104 Freiburg, Germany; ^2^ CIBSS - Centre for Integrative Biological Signalling Studies, University of Freiburg, 79104 Freiburg, Germany; ^3^ Department of Structural Cell Biology, Institute for Cell Biology and Neuroscience, Goethe University Frankfurt, 60438 Frankfurt, Germany; ^4^ Institute of Biochemistry II, Faculty of Medicine, Goethe University Frankfurt, 60590 Frankfurt, Germany; ^5^ Faculty of Biology, University of Freiburg, 79104 Freiburg, Germany

**Keywords:** ULK1, Autophagosome, Autophagy, SNARE, YKT6

## Abstract

Autophagy is a catabolic process during which cytosolic material is enwrapped in a newly formed double-membrane structure called the autophagosome, and subsequently targeted for degradation in the lytic compartment of the cell. The fusion of autophagosomes with the lytic compartment is a tightly regulated step and involves membrane-bound SNARE proteins. These play a crucial role as they promote lipid mixing and fusion of the opposing membranes. Among the SNARE proteins implicated in autophagy, the essential SNARE protein YKT6 is the only SNARE protein that is evolutionarily conserved from yeast to humans. Here, we show that alterations in YKT6 function, in both mammalian cells and nematodes, produce early and late autophagy defects that result in reduced survival. Moreover, mammalian autophagosomal YKT6 is phospho-regulated by the ULK1 kinase, preventing premature bundling with the lysosomal SNARE proteins and thereby inhibiting autophagosome–lysosome fusion. Together, our findings reveal that timely regulation of the YKT6 phosphorylation status is crucial throughout autophagy progression and cell survival.

## Teaser

**Summary:** The SNARE protein YKT6 plays a conserved role in autophagosome formation and fusion with lysosomes, which is controlled by the ULK1 kinase.

## INTRODUCTION

Macroautophagy, hereafter referred to as autophagy, is a highly conserved catabolic process in which cellular components, ranging from cytosolic proteins to whole organelles, are degraded in the lytic compartment of the cell (the vacuole in yeast or the lysosomes in mammals). Central to autophagy is the formation of a double-membrane vesicle, the autophagosome, around the cargo that is destined for degradation. Autophagic cargo can be taken up randomly during starvation conditions or in a selective manner with the help of cargo receptors. The closed and matured autophagosome then fuses with the lytic compartment, releasing the cargo material for degradation. The building blocks provided by this process can be recycled and allow the cell to undergo anabolic activities or generate energy (Hollenstein and Kraft, 2020).

Key in the final fusion step between the autophagosome and the lytic compartment are soluble N-ethylmaleimide sensitive factor attachment protein receptor (SNARE) proteins. SNARE proteins are classified according to a central interaction motif in their SNARE domain into R-SNAREs and Q-SNAREs, which are further subdivided into Qa-, Qb- and Qc-SNAREs (Fasshauer et al., 1998; Hong, 2005). During fusion, R- and Q-SNAREs from opposing membranes form a four α-helical bundle. The energy released during bundling into RQaQbQc complexes pulls the membranes into close proximity and allows lipid mixing and fusion of the membranes (Li et al., 2007). The fusion process is additionally supported by Rab GTPases and tethering complexes (D'Agostino et al., 2017).

In mammals, two SNARE complexes involved in autophagosome–lysosome fusion have been identified. These are the autophagosomal Qa-SNARE STX17 associated with the cytosolic Qbc-SNARE SNAP29 and the lysosomal R-SNARE VAMP8 (Itakura et al., 2012), or the autophagosomal R-SNARE YKT6 together with SNAP29 and the lysosomal Qa-SNARE STX7 (Matsui et al., 2018). Whether these two SNARE complexes act redundantly or are involved in different kinds of autophagosome–lysosome fusion processes is unclear. Their different degree of conservation suggests a possible independence in their roles (Kriegenburg et al., 2019; Matsui et al., 2018).

Among mammals and yeast, the autophagosomal protein YKT6 is the only SNARE conserved in yeast, where it also functions in autophagosome–vacuole fusion (Bas et al., 2018; Gao et al., 2018). YKT6 consists of an N-terminal longin domain, a C-terminal SNARE domain and a lipidation anchor region at the very C-terminus (Kriegenburg et al., 2019). Whereas most organelle-targeted SNARE proteins are integral membrane proteins, membrane anchoring of YKT6 is promoted by its C-terminal lipid modifications including farnesylation, palmitoylation and/or geranylgeranylation (Daste et al., 2015; Fukasawa et al., 2004; Shirakawa et al., 2020). That allows YKT6 an uncommon flexibility to localize to many different organelles, such as endosomes, autophagosomes, vacuoles and the Golgi (Bas et al., 2018; Dietrich et al., 2005; Hasegawa et al., 2003; Matsui et al., 2018). This, together with its ability to interact with nearly all Qa SNAREs (Matsui et al., 2018), allows YKT6 to participate in different intracellular trafficking pathways (Bas et al., 2018; Ungermann et al., 1999). This promiscuity with regard to binding partners and target membranes complicates the study of its pathway-specific interactors and regulation.

It has been shown that phosphorylation of YKT6 regulates its function (Barz et al., 2020; Gao et al., 2020; Karuna et al., 2020; Linnemannstöns et al., 2020; McGrath et al., 2021). In yeast, phosphorylation of the Ykt6 SNARE domain by Atg1 controls Ykt6 bundling with vacuolar SNARE proteins and thereby prevents premature fusion of nascent autophagosomes with the vacuole. Moreover, autophagosome formation is impaired (Barz et al., 2020; Gao et al., 2020). In mammals, phosphorylation of the YKT6 SNARE domain has been reported to induce a conformational switch in YKT6, which alters YKT6 membrane association and results in defects along the secretory pathway and potentially autophagy (Karuna et al., 2020; Linnemannstöns et al., 2020; McGrath et al., 2021).

Here, we show that the SNARE domain of mammalian YKT6 is phosphorylated by ULK1, which leads to an impairment of autophagosome formation and autophagosome–lysosome fusion. ULK1-dependent phosphorylation of YKT6 prevents its interaction with the Qbc-SNARE SNAP29 without affecting YKT6 membrane association. Importantly, timely phospho-regulation of YKT6 is critical during stress-related and prosurvival responses, such as overcoming mitochondrial damage. The prosurvival role of YKT6 was also confirmed in the multicellular model organism *Caenorhabditis elegans*. Besides its role in autophagosome formation and fusion, we demonstrate that *C. elegans* YKT-6 is required for the efficient removal of apoptotic cells in the germ line by LC3-associated phagocytosis (LAP), and is also regulated by phosphorylation.

These findings underline the importance of YKT6 function and phospho-regulation not only during autophagosome/phagosome fusion with the lytic compartment, but in addition highlight its evolutionary conserved relevance in allowing proper autophagy progression and sustaining cell viability.

## RESULTS

### YKT6 is a ULK1 kinase substrate

**Fig. 1.**
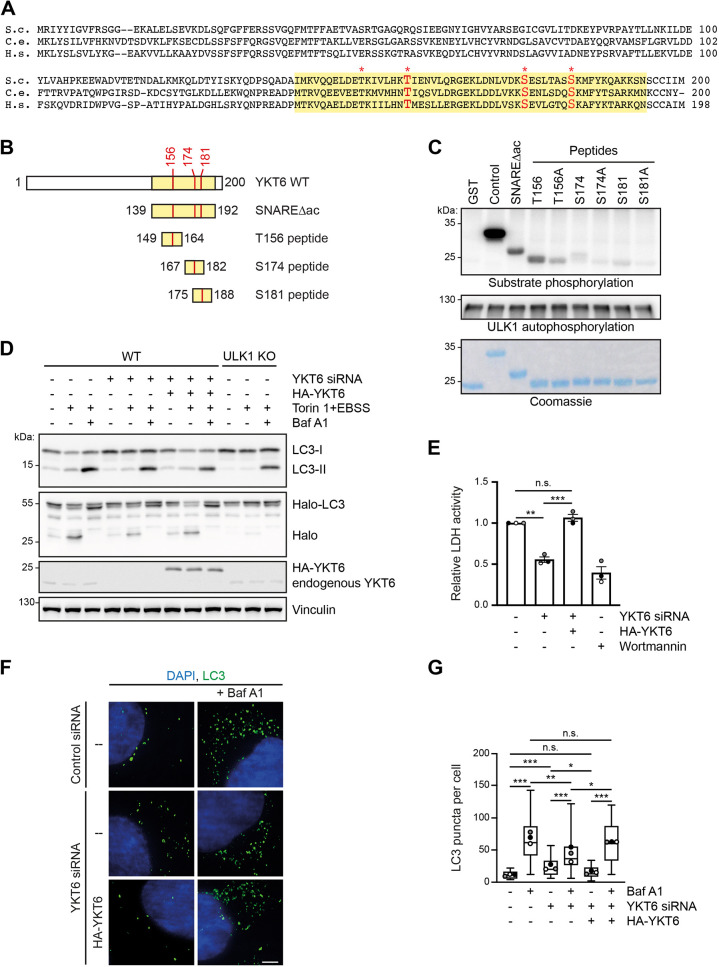
**YKT6 is phosphorylated by ULK1.** (A) Alignment of yeast (S.c., *Saccharomyces cerevisiae*), *C. elegans* (C.e.) and human (H.s.) YKT6 protein sequences. The SNARE domain is labelled in yellow, the conserved threonine and serine sites are marked by an asterisk, and the phosphorylation sites relevant to this study are marked in red. (B) Constructs used in the *in vitro* kinase assay shown in C. (C) GST, GST–Atg19 C-terminus (Control), GST–YKT6Δac (139–192 aa), and the GST–YKT6 peptides shown in B, and indicated alanine mutants thereof, were purified from *E. coli.* Purified GST fusion proteins were subjected to an *in vitro* kinase assay with recombinant ULK1. (D) HEK293T WT cells stably expressing Halo–LC3 and siRNA-resistant HA–YKT6 were transfected with YKT6 siRNA for 48 h to deplete endogenous YKT6. At 4 h prior to harvesting, 200 nM TMR HaloTag ligand was added to the cell medium for 10 min. The ligand was removed by washing two times with PBS. For the remaining time, the cells were grown either in DMEM or EBSS with 300 nM Torin 1 with or without 200 nM Bafilomycin A1 as indicated. (E) HEK293T WT cells or HEK293T cells stably expressing siRNA-resistant HA–YKT6 were transfected with YKT6 siRNA for 48 h. Cells were then treated for 4 h with EBSS, 300 nM Torin 1 and 200 nM Bafilomycin A1 as well as 200 nM wortmannin when indicated. Harvested cells were fractionated and the enzymatic activity of LDH was measured. The LDH activity in the membrane fraction compared to the total extract is represented as mean±s.e.m., with dots indicating the value of each biological replicate. Three independent biological replicates were performed. **P<0.01; ***P<0.001; n.s., not significant, *P*>0.05 (one-way ANOVA followed by a Bonferroni post-hoc test). (F,G) U2OS WT cells or U2OS cells stably expressing siRNA-resistant HA–YKT6 WT were transfected with either control siRNA or YKT6 siRNA for 48 h. The cells were grown in EBSS and 300 nM Torin 1 with or without 200 nM Bafilomycin A1, as indicated. Cells were then immunostained with anti-LC3 antibodies and DAPI (nucleus). The number of LC3 puncta from at least 30 cells was quantified and represented in a box and whisker plot (G). The box represents the 25–75th percentiles, and the median is indicated. The whiskers show the range. Dots indicate the mean of each biological replicate. *P<0.05, **P<0.01, ***P<0.001; n.s., not significant, *P*>0.05 (one-way ANOVA followed by a Bonferroni post-hoc test). Scale bar: 5 µm. One out of three independent biological replicates is shown in C, D and F.

Yeast and human YKT6 are required for autophagosome–vacuole fusion (Bas et al., 2018; Matsui et al., 2018). This fusion event is regulated by Atg1-dependent phosphorylation of Ykt6 in yeast, and phospho-mimetic mutations of Thr158, and Ser182 and/or Ser183 impair autophagosome fusion with the vacuole (Barz et al., 2020; Gao et al., 2020). Sequence alignment of the yeast Ykt6 SNARE domain with the human and the metazoan *C. elegans* sequences revealed that these sites are highly conserved, in addition to two further putative phosphorylation sites in the SNARE domain. These are Thr151, Thr158, Ser176 and Ser183 in yeast, corresponding to Thr149, Thr156, Ser174 and Ser181 in humans, and Thr152, Thr159, Ser177 and Ser184 in *C. elegans* (Fig. 1A). We therefore wondered whether the regulation of autophagosome–lytic compartment fusion is mechanistically conserved.

To address this question, we assessed whether mammalian YKT6 is a target of the human Atg1 homolog ULK1. We focused on Thr156 (yeast Thr158) and Ser181 (yeast Ser183), which are fusion-relevant in yeast (Barz et al., 2020; Gao et al., 2020). In addition, we analyzed Ser174 (yeast Ser176), for which an autophagy defect has been previously observed (McGrath et al., 2021) but with conflicting results reported (Karuna et al., 2020).

To determine whether ULK1 directly phosphorylates YKT6, we expressed GST fusion proteins containing the human YKT6 SNARE domain lacking the C-terminal acylation sites (SNAREΔac) or short amino acid peptides spanning either Thr156, Ser174 or Ser181 in *E. coli* (Fig. 1B). The isolated GST fusion constructs were subjected to *in vitro* phosphorylation using recombinant ULK1. Whereas the SNARE domain and the Thr156 peptide were readily phosphorylated, hardly any phosphorylation was observed for the Ser174 and Ser181 peptides (Fig. 1C). Alanine mutation of Thr156 (T156A) largely abolished *in vitro* phosphorylation by ULK1, but alanine mutation of Ser174 and Ser181 only led to a mild reduction. Together, these findings show that Thr156 in human YKT6, but not Ser174 and Ser181, is a direct target of the ULK1 kinase. We therefore mainly focused on Thr156 in subsequent experiments.

### YKT6 phosphorylation impairs the autophagy flux

To study the effect of YKT6 phosphorylation in autophagy, we employed siRNA-resistant YKT6 constructs in cells depleted of endogenous YKT6 by siRNA (Fig. S1A). To validate our setup, we monitored autophagy flux in YKT6 siRNA cells using the pulse-chasable reporter Halo-LC3B (LC3B is also known as MAP1LC3B, hereafter LC3) (Yim et al., 2022), and found that less free Halo was generated in YKT6 siRNA-treated cells compared to control siRNA-treated cells, confirming that YKT6 is required for efficient autophagy flux (Fig. 1D; Fig. S1B; Matsui et al., 2018). Similarly, we noticed a decrease in the LC3-II to LC3-I ratio in cells depleted for YKT6, after autophagy induction by incubation in Earle's balanced salts solution (EBSS) and addition of the mTOR inhibitor Torin 1 (Fig. 1D; Fig. S1C). Notably, additional treatment with the lysosomal fusion inhibitor Bafilomycin A1 only led to a partial accumulation of LC3-II in YKT6 knockdown cells (Fig. 1D; Fig. S1C). As LC3-II represents the lipidated form of LC3 and is a hallmark of autophagosome biogenesis, this lack of LC3-II accumulation indicates that YKT6 is required also for autophagosome formation (Mizushima and Yoshimori, 2007). As YKT6 has previously been described to function in autophagosome–lysosome fusion (Matsui et al., 2018), but its role in autophagosome formation has not been addressed, we substantiated these findings further by using the lactate dehydrogenase (LDH) sequestration assay (Seglen et al., 2015). The LDH assay monitors the uptake of cytosolic LDH into autophagosomes by bulk autophagy and therefore specifically assesses the autophagosome formation step. The engulfed LDH amount is then measured in the membrane fraction of Bafilomycin A1-treated cells. Cells depleted for endogenous YKT6 showed a decrease in LDH activity in the membrane fraction compared to control cells (Fig. 1E), further supporting that they have an autophagosome formation defect. Although autophagosome biogenesis is impaired in the absence of YKT6, we observed an enrichment of autophagosomes in the cytosol of YKT6-knockdown cells (Fig. 1F,G), which also supports that autophagosome–lysosome fusion is affected. Expression of HA-tagged YKT6 in cells depleted for YKT6 complemented both the autophagosome formation and fusion defects, supporting that the impairments in autophagy are YKT6 dependent (Fig. 1D–G; Fig. S1B,C).

**Fig. 2.**
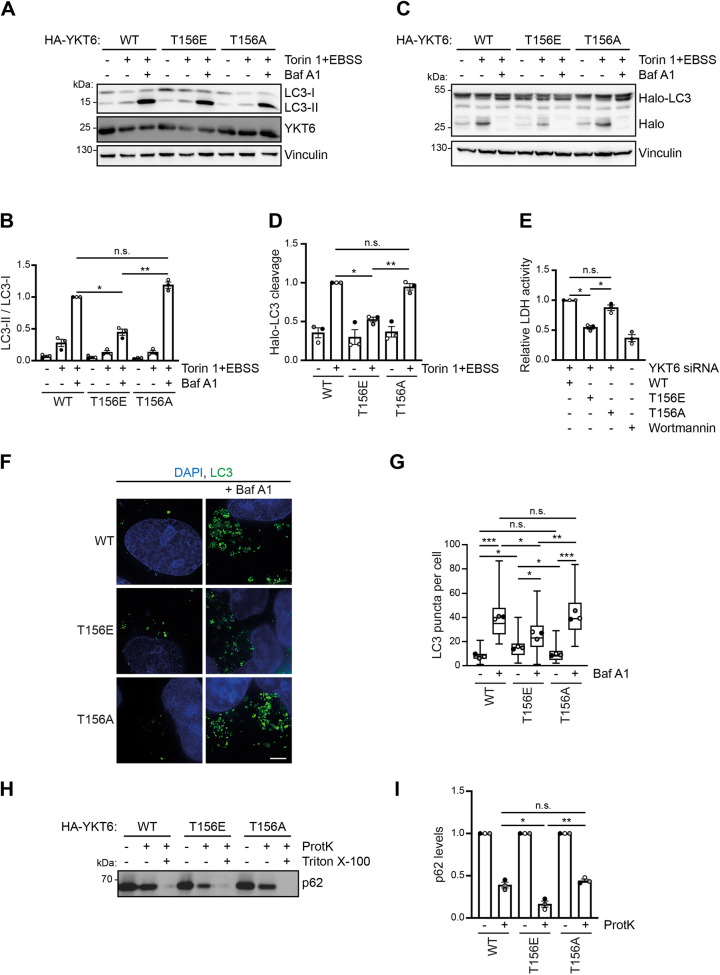
**YKT6 phosphorylation inhibits autophagy flux and autophagosome formation.** (A,B) HEK293T cells stably expressing siRNA-resistant HA–YKT6-WT, HA–YKT6-T156E or HA–YKT6-T156A were transfected with YKT6 siRNA for 48 h and grown either in DMEM or EBSS with 300 nM Torin 1 with or without 200 nM Bafilomycin A1 as indicated. LC3 lipidation was analyzed by western blotting. Densitometric quantification of the LC3-II to LC3-I ratio relative to vinculin was performed and is shown as means±s.e.m., with dots indicating the value of each biological replicate (B). *P<0.05, **P<0.01, n.s., not significant, *P*>0.05 (one-way ANOVA followed by a Bonferroni post-hoc test). (C,D) Cells from A additionally stably expressing Halo–LC3 were treated with 200 nM TMR HaloTag ligand for 10 min. The ligand was removed by washing two times with PBS. Subsequently, the cells were grown for 4 h either in DMEM or EBSS with 300 nM Torin 1 with or without 200 nM Bafilomycin A1 as indicated. Densitometric quantification of cleaved HaloTag relative to vinculin (D) was performed and is shown as means±s.e.m., with dots indicating the value of each biological replicate. *P<0.05; **P<0.01; n.s., not significant, *P*>0.05 (one-way ANOVA followed by a Bonferroni post-hoc test). (E) Cells from A were treated for 4 h with EBSS, 300 nM Torin 1 and 200 nM Bafilomycin A1 as well as 200 nM wortmannin when indicated. Harvested cells were lysed and fractionated and the enzymatic activity of LDH was measured. The LDH activity in the membrane fraction compared to the total extract is represented as means±s.e.m., with dots indicating the value of each biological replicate. Three independent biological replicates were performed. *P<0.05; n.s., not significant, *P*>0.05 (one-way ANOVA followed by a Bonferroni post-hoc test). (F,G) U2OS cells stably expressing the indicated siRNA-resistant HA–YKT6 constructs were transfected with YKT6 siRNA for 48 h. The cells were grown in EBSS and 300 nM Torin 1 with or without 200 nM Bafilomycin A1 as indicated. Subsequently, the cells were immunostained with anti-LC3 antibodies and DAPI (nucleus). The number of LC3 puncta from at least 30 cells was quantified and represented in a box and whisker plot (G). The box represents the 25–75th percentiles, and the median is indicated. The whiskers show the range. Dots indicate the mean of each biological replicate. *P<0.05, **P<0.01, ***P<0.001; n.s., not significant, *P*>0.05 (one-way ANOVA followed by a Bonferroni post-hoc test). Scale bar: 5 µm. (H,I) HEK293T cells stably expressing siRNA-resistant HA–YKT6-WT, HA–YKT6-T156E or HA–YKT6-T156A were transfected with YKT6 siRNA for 48 h. The cells were grown in EBSS medium with 300 nM Torin 1 for 2 h. After lysis, membrane fractions were subjected to proteinase K (ProtK) and Triton X-100 treatment as indicated, and analyzed by anti-p62 western blotting. Densitometric quantification of p62 protein levels normalized to the control sample is shown, represented as means±s.e.m., with dots indicating the value of each biological replicate (I). Owing to the absence of a western blot signal, the samples treated with both proteinase K and Triton X-100 were not quantified. *P<0.05, **P<0.01; n.s., not significant, *P*>0.05 (one-way ANOVA followed by a Bonferroni post-hoc test). One out of three independent biological replicates is shown in A, C, F and H.

Next, we tested whether non-phosphorylatable alanine (T156A) and phosphorylation-mimicking glutamate (T156E) YKT6 mutants also complemented the lack of YKT6, and how phosphorylation-mimicking on Thr156 affects the autophagy flux, by stably expressing HA-tagged YKT6 wild type (WT), HA-tagged non-phosphorylatable alanine (T156A) and HA-tagged phosphorylation-mimicking glutamate (T156E) mutants in HEK293T cells depleted for endogenous YKT6. Autophagy induction resulted in a slight increase of LC3 lipidation compared to basal growth conditions in WT YKT6-containing cells, whereas the addition of Bafilomycin A1 resulted in a strong increase of the LC3-II to LC3-I ratio, as expected. Whereas the HA–YKT6-T156A mutant showed similar LC3 lipidation to that in HA–YKT6-WT containing cells, the HA–YKT6-T156E mutant showed a decreased ratio of LC3-II to LC3-I, indicating an impaired autophagy flux (Fig. 2A,B). Similar results were obtained when monitoring Halo-LC3 cleavage (Fig. 2C,D) or LDH sequestration (Fig. 2E), suggesting that, similar to YKT6 depletion, the HA–YKT6-T156E mutant results in a defect in autophagosome formation.

In addition, LC3 puncta formation was decreased in the HA–YKT6-T156E mutant upon Torin 1 and Bafilomycin A1 treatment compared to HA–YKT6-WT- and HA–YKT6-T156A-containing cells, which further supports an impairment in the autophagy flux of the phospho-mimetic HA–YKT6-T156E mutant (Fig. 2F,G). Importantly, in Torin 1-treated cells, in which fusion can take place normally, the phospho-mimetic HA–YKT6-T156E mutant showed an increase in the number of LC3 puncta compared to HA–YKT6-WT- and HA–YKT6-T156A-containing cells, suggesting that although autophagosome formation is decreased, the autophagosomes that form fail to fuse with lysosomes, resulting in their accumulation.

To test whether autophagosome closure is affected in the YKT6 mutants, we performed a protease protection assay and monitored the sensitivity of the autophagy cargo protein p62 (also known as SQSTM1) to proteinase K by western blotting. Whereas p62 in sealed autophagosomes is protected, defects in autophagosome formation will render it sensitive to exogenous protease addition. In HEK293T cells expressing HA–YKT6-WT, p62 was partially protected from proteinase K, as expected (Fig. 2H; Zellner et al., 2021). Similar protection was observed in the HA–YKT6-T156A mutant; however, in the phospho-mimetic HA–YKT6-T156E mutant p62 showed higher sensitivity to proteinase K treatment, suggesting a failure in autophagosome closure (Fig. 2H,I).

Together, these results indicate that YKT6 functions not only in late steps of autophagy but also during autophagosome formation, and that these functions are regulated by ULK1-mediated phosphorylation.

### Thr156 phosphorylation and ULK1 do not affect YKT6 membrane association

**Fig. 3.**
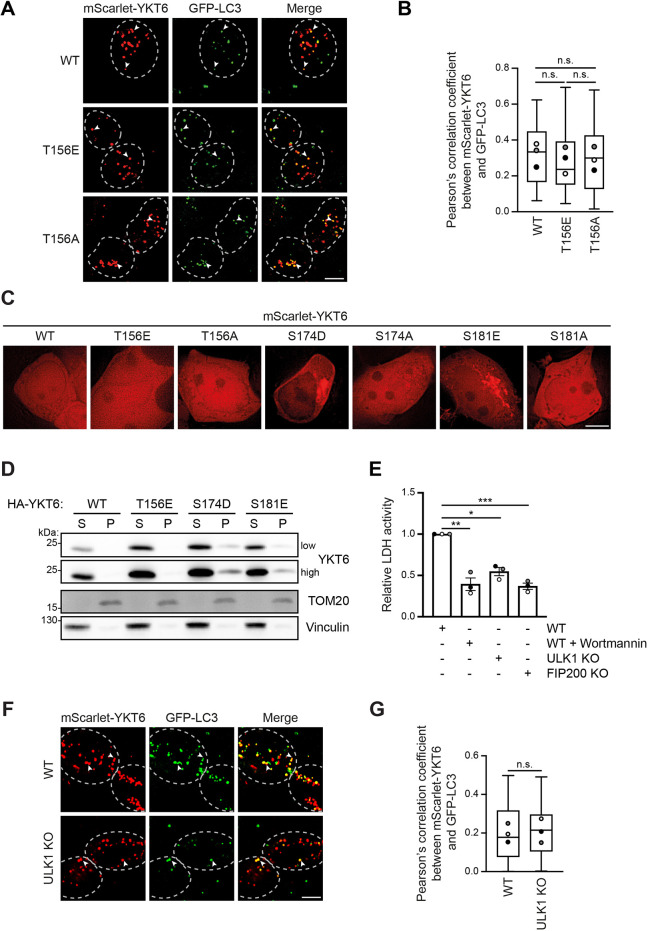
**YKT6 phosphorylated at T156 shows normal membrane association.** (A,B) YKT6 siRNA-treated HEK293T cells stably expressing GFP–LC3 were transiently transfected with the indicated siRNA-resistant mScarlet–YKT6 constructs for 24 h and treated for 4 h with EBSS and 300 nM Torin 1. After 15 min permeabilization with 50 µg/ml of digitonin, cells were visualized by fluorescence microscopy. Arrowheads highlight colocalization between YKT6 and LC3; cells are outlined by dashed lines. Colocalization of mScarlet–YKT6 and GFP–LC3 puncta was quantified in >30 cells and represented in a box and whisker plot (B). The box represents the 25–75th percentiles, and the median is indicated. The whiskers show the range. Dots indicate the mean of each biological replicate. n.s., not significant, *P*>0.05 (one-way ANOVA followed by a Bonferroni post-hoc test). Scale bar: 5 µm. (C) HEK293T cells transiently expressing the indicated mScarlet-YKT6 constructs were visualized by fluorescence microscopy. Scale bar: 5 µm. (D) HEK293T cells stably expressing the indicated siRNA-resistant HA–YKT6 constructs were treated with YKT6 siRNA for 48 h, lysed and separated into a cytosolic fraction (S) and a 100,000 ***g*** membrane pellet (P). The samples were analyzed by western blotting using anti-YKT6, anti-TOM20 (membrane marker) and anti-vinculin (cytosolic marker) antibodies. Low and high indicate the level of exposure. (E) HEK293T WT, ULK1 KO or FIP200 KO cells were treated for 4 h with EBSS, 300 nM Torin 1 and 200 nM Bafilomycin A1, as well as 200 nM wortmannin when indicated. Harvested cells were lysed and fractionated and the enzymatic activity of LDH was measured. The LDH activity in the membrane fraction compared to the total extract is represented as means±s.e.m., with dots indicating the value of each biological replicate. Three independent biological replicates were performed. *P<0.05; **P<0.01; ***P<0.001 (one-way ANOVA followed by a Bonferroni post-hoc test). (F,G) HEK293T WT or ULK1 KO cells stably expressing GFP-LC3 were treated as in A. Arrowheads highlight colocalization between YKT6 and LC3; cells are outlined by dashed lines. Colocalization of mScarlet–YKT6 and GFP–LC3 puncta was quantified in >30 cells and represented in a box and whisker plot (G). The box represents the 25–75th percentiles, and the median is indicated. The whiskers show the range. Dots indicate the mean of each biological replicate. n.s., not significant, *P*>0.05 (unpaired two-tailed *t*-test assuming unequal variances). Scale bar: 5 µm. Images in A, C, D and F are representative of three independent biological replicates.

YKT6 membrane association occurs via a lipid anchor (Shirakawa et al., 2020). Phosphorylation events in the SNARE domain of YKT6 have been proposed to regulate its membrane binding (Karuna et al., 2020; Linnemannstöns et al., 2020; McGrath et al., 2021). We therefore asked whether the autophagy defects observed in the phospho-mimetic T156E mutant result from an impaired recruitment to autophagosomal membranes. We analyzed the colocalization of YKT6 with autophagosomes by fluorescence microscopy in HEK293T cells transiently expressing mScarlet–YKT6-WT, mScarlet–YKT6-T156E or mScarlet–YKT6-T156A, as well as the autophagosomal marker protein GFP–LC3. As YKT6 and LC3 are also present in the cytosol, the cells were permeabilized prior to imaging, in order to remove the cytosolic background signal. mScarlet–YKT6-WT and the T156E or T156A mutant variants were present in defined punctate structures that partially colocalized with GFP–LC3, as previously described for the YKT6-WT protein (Fig. 3A,B; Matsui et al., 2018). The GFP–LC3 puncta observed were confirmed to be autophagosomes, as treatment with wortmannin drastically reduced their abundance and colocalization with mScarlet–YKT6 (Fig. S2A,B). The degree of colocalization was similar in all mScarlet–YKT6 variants, indicating that YKT6 association with autophagosomes is independent of the phosphorylation status at Thr156. Although phosphorylation at Thr156 in the YKT6 SNARE domain does not affect autophagosomal membrane association, it had been previously shown that the phospho-status of the SNARE domain can influence its general membrane localization. However, we did not observe any enhanced membrane association of the mScarlet–YKT6-T156E mutant, whereas the phospho-mimetic mScarlet–YKT6-S174D showed an increase in membrane localization, as previously reported (Fig. 3C; Fig. S2C; McGrath et al., 2021). Also the single mScarlet–YKT6-S181E variant was enriched on membranes (Fig. 3C), similar to a reported triple YKT6 phospho-mutant containing this site (Karuna et al., 2020; Linnemannstöns et al., 2020). In agreement with these microscopy results, membrane fractionation experiments revealed no enrichment of HA–YKT6-T156E at membranes (Fig. 3D). Together, these findings suggest that YKT6 phosphorylation on Thr156 does not alter its membrane localization properties.

In yeast, it has been suggested that Ykt6 association with autophagosomal membranes depends on the presence of the Atg1 kinase (Gao et al., 2020). To test whether mammalian YKT6 also requires ULK1 for the association with autophagosomes, we monitored mScarlet–YKT6-WT colocalization with GFP–LC3 in WT and ULK1 knockout (KO) HEK293T cells. ULK1 KO cells can still form autophagosomes although to a lesser degree than WT cells (McAlpine et al., 2013). Hence, autophagy in ULK1 KO cells is not completely defective compared to FIP200 KO cells. FIP200 is a member of the ULK1 complex and has been shown previously to have a complete autophagy defect upon deletion (Fig. 1D, Fig. S1B,C, Fig. 3E; Fig. S2D–G; McAlpine et al., 2013). As no difference of YKT6 colocalization with GFP–LC3 in the presence and absence of ULK1 was observed (Fig. 3F,G), we conclude that neither ULK1 nor phosphorylation at Thr156 alters the association of YKT6 with autophagosomal membranes. Therefore, phosphorylation at Thr156 must regulate YKT6 function on the autophagosomal membrane by other means.

### YKT6 phosphorylation affects autophagosome–lysosome fusion

Bafilomycin A1 inhibits autophagosome–lysosome fusion and therefore results in an accumulation of autophagosomes in the cytosol (Yamamoto et al., 1998). LC3 puncta formation was decreased in Bafilomycin A1-treated HA–YKT6-T156E cells compared to HA–YKT6-WT cells, suggesting a defect in autophagosome formation (Fig. 2F,G). However, in the absence of Bafilomycin A1 treatment, LC3 puncta accumulated in HA-YKT6-T156E cells, suggesting an impairment not only in autophagosome formation, but also in their fusion with lysosomes (Fig. 2F,G).

**Fig. 4.**
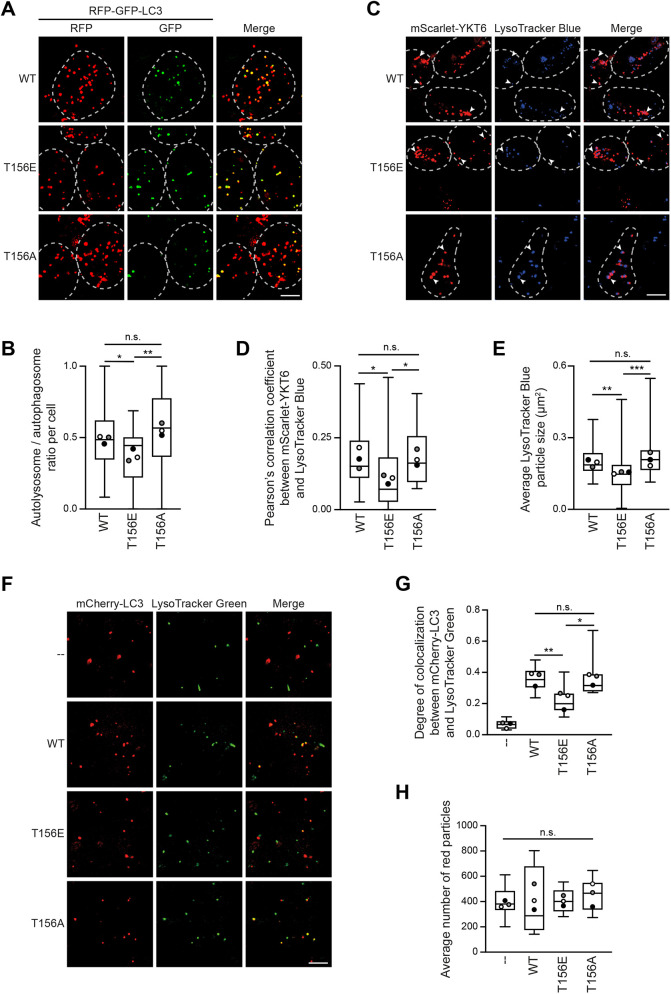
**YKT6 phosphorylation prevents autophagosome–lysosome fusion.** (A,B) HEK293T cells stably expressing siRNA-resistant HA–YKT6-WT, HA–YKT6-T156E or HA–YKT6-T156A were transfected with YKT6 siRNA for 48 h. The cells were transiently transfected with RFP–GFP–LC3 for 24 h and treated for 4 h with EBSS and 300 nM Torin 1. After 15 min permeabilization with 50 µg/ml of digitonin, cells were visualized by fluorescence microscopy. Cells are outlined by dashed lines. The ratio of autolysosomes (red-only puncta) to autophagosomes (red and green puncta) was quantified in >30 cells and represented in a box and whisker plot (B). The box represents the 25–75th percentiles, and the median is indicated. The whiskers show the range. Dots indicate the mean of each biological replicate. *P<0.05; **P<0.01; n.s., not significant, *P*>0.05 (one-way ANOVA followed by a Bonferroni post-hoc test). Scale bar: 5 µm. (C–E) YKT6 siRNA treated HEK293T cells were transiently transfected with the indicated mScarlet–YKT6 constructs. The cells were treated for 4 h with EBSS and 300 nM Torin 1 and for the last 45 min with 100 nM LysoTracker Blue. After 15 min permeabilization with 50 µg/ml of digitonin, cells were visualized by fluorescence microscopy. Arrowheads highlight mScarlet–YKT6-positive structures; cells are outlined by dashed lines (C). Colocalization of mScarlet–YKT6 and LysoTracker Blue puncta (D) and the average size of LysoTracker Blue puncta (E) were quantified in >30 cells and represented in a box and whisker plot. The box represents the 25–75th percentiles, and the median is indicated. The whiskers show the range. Dots indicate the mean of each biological replicate. *P<0.05; **P<0.01; ***P<0.001; n.s., not significant, *P*>0.05 (one-way ANOVA followed by a Bonferroni post-hoc test). Scale bar: 5 µm. (F–H) Autophagosomes were enriched from Torin 1-treated HEK293T cells stably expressing the different YKT6 mutant variants as well as mCherry–LC3. Lysosomes were enriched from Torin 1-treated FIP200 KO cells stained with LysoTracker Green. The cytosol was obtained from HEK293T WT cells. To reconstitute fusion *in vitro*, the autophagosomal, the lysosomal and the cytosolic fraction were mixed and supplemented with an ATP regeneration system. Fusion, represented by the colocalization between red (autophagosomes) and green (lysosomes) particles was then analyzed by fluorescent microscopy (F) and quantified. The box and whiskers plot of the colocalization degree is depicted in (G). The box represents the 25–75th percentiles, and the median is indicated. The whiskers show the range. Dots indicate the mean of each biological replicate. The average number of red particles was measured to ensure all samples had a similar number of autophagosomes and is represented in H as a box and whiskers plot, with dots indicating the mean of each biological replicate *P<0.05; **P<0.01; n.s., not significant, *P*>0.05 (one-way ANOVA followed by a Bonferroni post-hoc test). Scale bar: 5 µm. Images in A, C and F are representative of three independent biological replicates.

To assess the efficiency of autophagosome–lysosome fusion in more detail, we monitored RFP–GFP–LC3 by fluorescence microscopy. As the GFP signal is quenched by the acidic environment of the lysosome whereas the fluorescence of RFP remains stable, this reporter construct is used to distinguish unfused autophagosomes (green/red=yellow) from fused autolysosomes (red) (Kimura et al., 2007). In agreement with a defect in autophagosome–lysosome fusion, HEK293T cells expressing the HA–YKT6-T156E variant showed a smaller ratio of autolysosomes to autophagosomes compared to the ratio observed in HA–YKT6-WT- and HA–YKT6-T156A-expressing cells, pointing to a defective turnover of formed autophagosomes (Fig. 4A,B). In addition, for the membrane-enriched HA–YKT6-S174D mutant we noticed an accumulation of autophagosomes and a reduced ratio of autolysosomes to autophagosomes (Fig. S3A,B), pointing to late autophagy defects, whereas previously only an early impairment in autophagy had been reported (McGrath et al., 2021). In contrast, HA–YKT6-S181E did not affect autophagy (Fig. S3A,B).

As the autophagosomal SNARE YKT6 will end up at the lysosomal membrane after autophagosome–lysosome fusion, we analyzed the colocalization of lysosomes stained with LysoTracker Blue and mScarlet–YKT6 (Fig. 4C,D). The colocalization between both structures was significantly reduced in HEK293T cells expressing the phospho-mimetic mScarlet–YKT6-T156E variant compared to the colocalization in cells expressing mScarlet–YKT6-WT or the mScarlet–YKT6-T156A mutant, further supporting that phosphorylation on YKT6 at Thr156 impairs autophagosome–lysosome fusion. Bafilomycin A1 treatment resulted in an almost complete loss of colocalization of mScarlet–YKT6-WT with lysosomes (Fig. S3C,D), ruling out a possible direct recruitment of YKT6 to lysosomes (Takáts et al., 2018).

Upon fusion of autophagosomes with lysosomes, autolysosomes increase in size in flies (Mauvezin et al., 2015). Thus, the average size of lysosomal structures, including both lysosomes and autolysosomes, can be used as an indirect measure for autophagosome–lysosome fusion. Indeed, it was also the case in mammals that LysoTracker Blue-positive RFP–GFP–LC3 puncta (autolysosomes, arrows) were larger in size than LysoTracker Blue-positive puncta that did not colocalize with RFP–GFP–LC3 (lysosomes, arrowheads, Fig. S3E,F). In line with a defect in autophagosome–lysosome fusion, HEK293T cells expressing the mScarlet–YKT6-T156E variant showed a smaller average size of LysoTracker Blue-positive structures compared to those expressing mScarlet–YKT6-WT and the mScarlet–YKT6-T156A mutant (Fig. 4E).

To uncouple the autophagosome formation from the autophagosome–lysosome fusion step, we reconstituted autophagosome–lysosome fusion *in vitro*. We enriched autophagosomes from Torin 1-treated HEK293T cells stably expressing the different YKT6 mutant variants as well as mCherry–LC3, and lysosomes from Torin 1-treated FIP200 KO cells stained with LysoTracker Green. The cytosol was obtained from HEK293T WT cells by using the 20,000 ***g*** supernatant. To reconstitute fusion, the autophagosomal, the lysosomal and the cytosolic fraction were mixed and supplemented with an ATP regeneration system. Successful fusion was assessed by the colocalization of mCherry–LC3 and LysoTracker Green. As a negative control, the autophagosomal and lysosomal fractions were mixed without cytosol and ATP. In each set-up, a similar number of mCherry–LC3 particles was used. Whereas substantial fusion was seen when autophagosomes were obtained from cells expressing HA–YKT6-WT or HA–YKT6-T156A, autophagosomes from the HA–YKT6-T156E mutant showed a significant fusion defect (Fig. 4F–H).

Taken together, these findings show that YKT6 phosphorylation at Thr156 impairs autophagosome–lysosome fusion.

### YKT6 phosphorylation at Thr156 impairs SNARE bundling

**Fig. 5.**
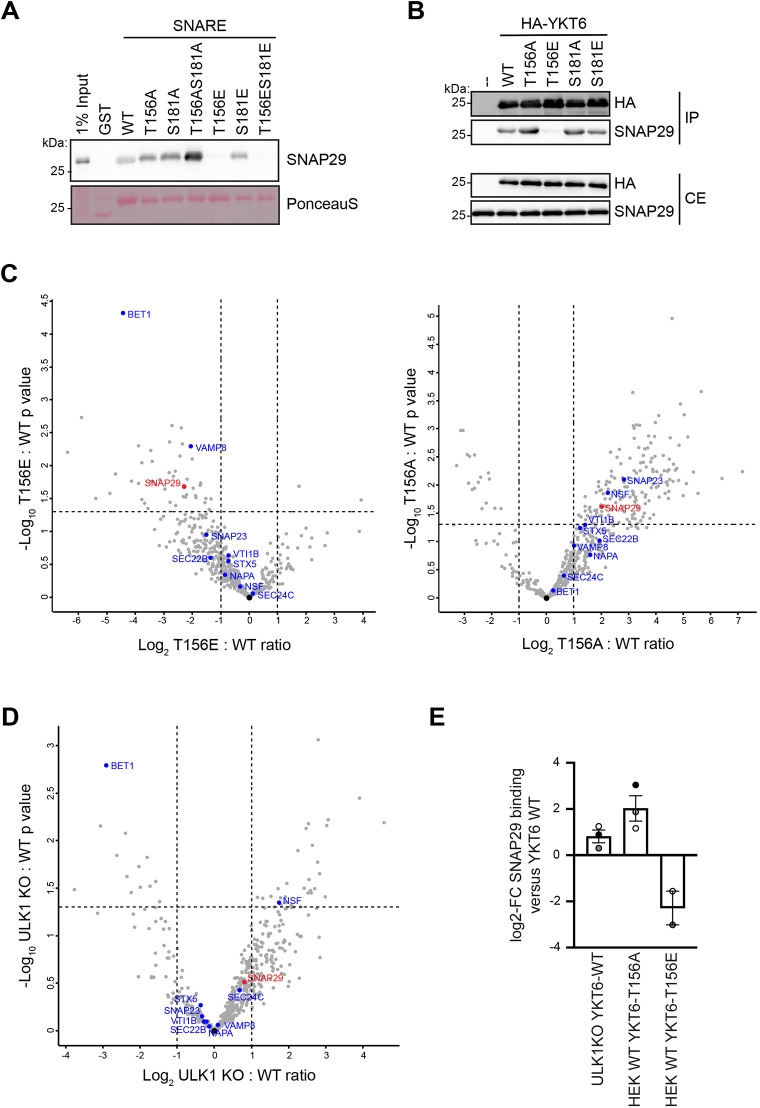
**YKT6 phosphorylation causes defective SNARE bundling.** (A) GST and the indicated GST–YKT6 SNARE domain variants expressed in *E. coli* were immobilized on GSH–Sepharose beads and subjected to an *in vitro* pulldown assay with crude cell extracts from HEK293T cells. Co-precipitating proteins were analyzed by anti-SNAP29 western blotting. One out of three independent biological replicates is shown. (B) YKT6 siRNA-treated HEK293T cells stably expressing the indicated HA–YKT6 constructs were treated for 2 h with EBSS and 300 nM Torin 1 and subjected to immunoprecipitation using HA–agarose beads, followed by western blot analysis. IP, immunoprecipitation; CE, crude extract. Images are representative of three independent biological replicates. (C,D) YKT6 siRNA-treated HEK293T WT or ULK1 KO cells stably expressing the indicated HA–YKT6 constructs were treated for 2 h with EBSS and 300 nM Torin 1 and subjected to immunoprecipitation followed by mass spectrometry. Volcano plots comparing the ratio of co-immunoprecipitated proteins in HA–YKT6-T156E to HA–YKT6-WT (C, left), HA–YKT6-T156A to HA–YKT6-WT (C, right) or HA–YKT6-WT in ULK1 KO to WT cells (D) are shown. Three independent biological replicates were performed. (E) The fold change of SNAP29 binding to YKT6 in the indicated strains compared to HEK293T WT cells stably expressing HA–YKT6-WT is shown as means±s.e.m., with dots indicating the value of each biological replicate. Three independent biological replicates were performed.

Phosphorylation of yeast Ykt6 in its SNARE domain prevents Ykt6 binding to the vacuolar SNARE proteins Vti1 and Vam3 and therefore inhibits autophagosome–vacuole fusion (Barz et al., 2020). In mammals, autophagosomal YKT6 forms a complex with the SNAREs STX7 and SNAP29 during autophagosome fusion with lysosomes. To assess whether phosphorylation of mammalian YKT6 prevents its interaction with these SNARE proteins, we analyzed the YKT6 interaction with SNAP29 *in vitro*. The recombinant GST-tagged SNARE domain of YKT6 or its phospho-mutant variants lacking the C-terminal acylation sites were immobilized on beads and incubated with cell extract from HEK293T cells. Whereas GST–YKT6-WT and GST–YKT6-T156A were proficient in co-precipitating SNAP29 from cell lysates, this interaction was absent in the GST–YKT6-T156E mutant (Fig. 5A). GST–YKT6-S181E, which corresponds to the conserved serine site crucial for SNARE interaction in yeast, however, showed normal binding to SNAP29, reconfirming the predominant role of Thr156 in mammals.

We substantiated these findings *in vivo* by co-immunoprecipitation experiments. In line with our *in vitro* findings, full-length HA–YKT6-WT proficiently co-precipitated SNAP29 in HEK293T cells, whereas the SNAP29 interaction was lost in the HA–YKT6-T156E mutant (Fig. 5B). On the other hand, the T156A mutation in HA–YKT6 enhanced the association with SNAP29. Similar to what was seen in the *in vitro* data, the SNAP29–YKT6 interaction was unaffected by the Ser181 mutation (Fig. 5B). Mass spectrometry confirmed our findings (Fig. 5C) and furthermore showed a slightly increased binding of YKT6 to SNAP29 in ULK1 KO cells (Fig. 5D,E), further supporting that ULK1 acts as a negative regulator of this interaction.

In summary, our data suggests that the phosphorylation of YKT6 at Thr156 in its SNARE domain abolishes the interaction with other SNARE proteins and therefore SNARE bundling and subsequent autophagosome–lysosome fusion.

### Prosurvival mitophagy is impaired by YKT6 phosphorylation

**Fig. 6.**
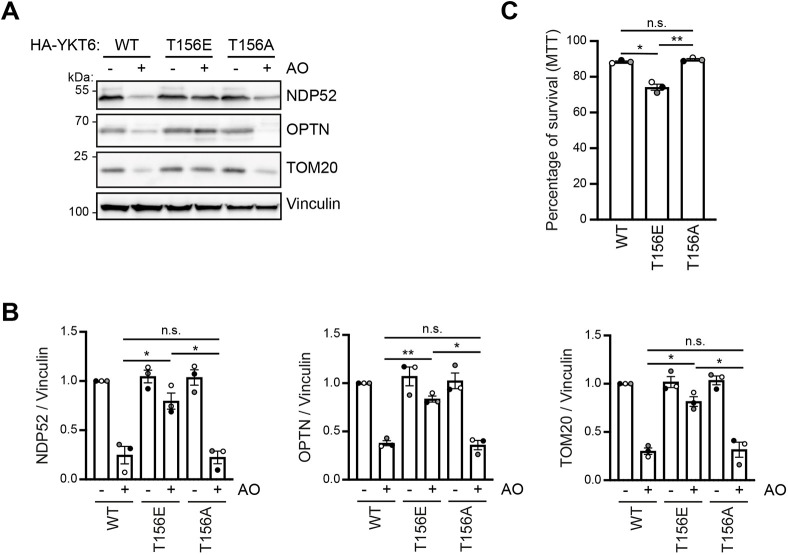
**YKT6 phosphorylation inhibits prosurvival autophagy.** (A,B) YKT6 siRNA-treated HEK293T cells stably expressing the indicated HA–YKT6 constructs were treated for 8 h with 10 µM antimycin A and oligomycin (AO), as indicated. Samples were analyzed by western blotting. Images are representative of three independent biological replicates. Densitometric quantification of NDP52 (left), OPTN (middle) or TOM20 (right) levels relative to vinculin is shown as means±s.e.m., with dots indicating the value of each biological replicate (B). *P<0.05; **P<0.01; n.s., not significant, *P*>0.05 (one-way ANOVA followed by a Bonferroni post-hoc test). (C) YKT6 siRNA-treated HEK293T cells stably expressing the indicated HA–YKT6 constructs were treated for 72 h with 10 µM antimycin A and oligomycin. Cell survival was analyzed by an MTT assay and expressed as percentage of cell survival in comparison to untreated samples, which were set to 100% survival. Results are shown as means±s.e.m., with dots indicating the value of each biological replicate. Three independent biological replicates were performed *P<0.05; **P<0.01; n.s., not significant, *P*>0.05 (one-way ANOVA followed by a Bonferroni post-hoc test).

As phospho-mimetic YKT6-T156E showed defects in bulk autophagy (Fig. 2A–E), we next asked whether ULK1-dependent phosphorylation of YKT6 also controls mitophagy, the selective degradation of damaged mitochondria. We induced parkin-dependent mitophagy using antimycin A and oligomycin (AO) in HEK293T cells expressing HA–YKT6-WT, HA–YKT6-T156A or HA–YKT6-T156E. Mitochondrial turnover was assessed by monitoring the degradation of the mitochondrial protein TOM20 (also known as TOMM20) as well as the mitophagy receptors NDP52 (also known as CALCOCO2) and optineurin (OPTN) by western blotting (Pickles et al., 2018). We saw a near complete block in the degradation of TOM20 and the mitophagy receptors in cells expressing HA–YKT6-T156E, whereas in HA–YKT6-WT- and HA–YKT6-T156A-containing cells these proteins were turned over efficiently (Fig. 6A,B). Furthermore, we observed a decrease in cell viability of HA–YKT6-T156E mutant cells compared to HA–YKT6-WT- and HA–YKT6-T156A-expressing cells, indicating that impaired mitochondria clearance in YKT6-T156E containing cells negatively affects cell survival upon mitochondrial stress induction (Fig. 6C). These findings suggest that timely controlled ULK1-dependent phosphorylation of YKT6 at Thr156 is crucial for selective autophagy and cell survival.

### YKT-6 is required for early and late steps of autophagy in *C. elegans*

**Fig. 7.**
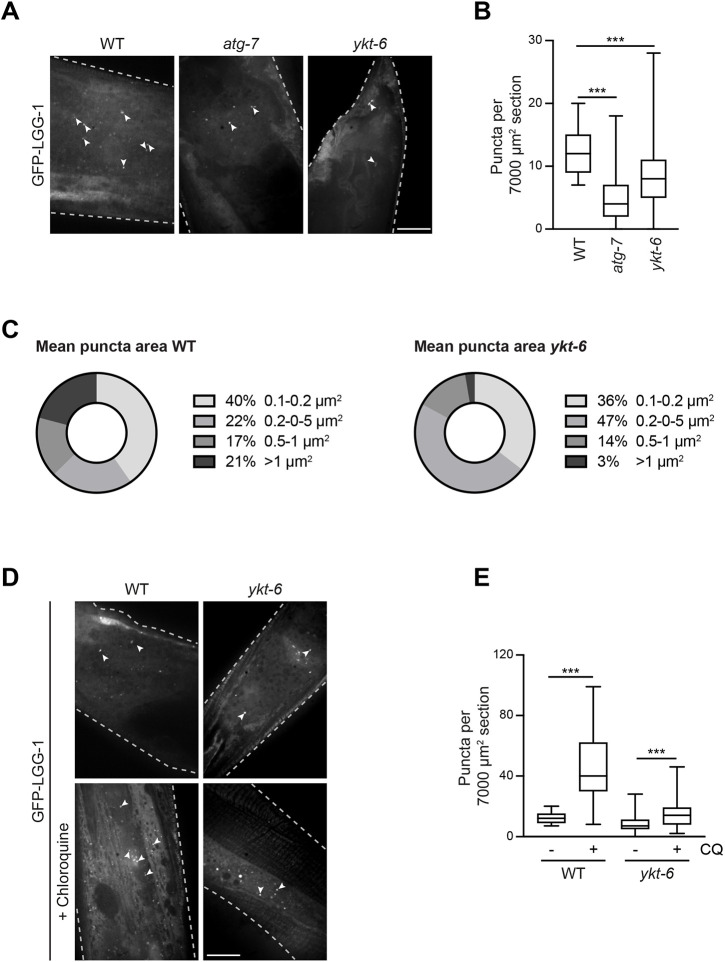
**YKT-6 is required for early steps in autophagy in *C. elegans.*** (A,B) Post-hatching worms were fed with RNAi to deplete the indicated proteins or with empty vector as a WT control. At the 1-day-old adult stage, the epidermis was visualized by fluorescent microscopy. The number of GFP–LGG-1 puncta (arrowheads in A) per area was quantified in 100 tail and pharynx sections and represented in a box and whiskers plot (B). The box represents the 25–75th percentiles, and the median is indicated. The whiskers show the range. ***P<0.001 (one-way ANOVA followed by a Bonferroni post-hoc test). Scale bar: 20 µm. Images are representative of three independent biological replicates. (C) The size of 100 GFP–LGG-1 puncta from WT or *ykt-6* knockdown worms from A was analyzed. (D,E) Animals treated as in (A) were subjected to 5 mM chloroquine treatment for 24 h, as indicated. The number of GFP–LGG-1 puncta per area (arrowheads) was quantified in 100 tail and pharynx sections and represented in a box and whisker plot (E). The box represents the 25–75th percentiles, and the median is indicated. The whiskers show the range. ***P<0.001 (one-way ANOVA followed by a Bonferroni post-hoc test). Scale bar: 20 µm. Images in A and D are representative of three independent biological replicates; dashed lines highlight the edge of the gonad arm.

*C. elegans* YKT-6 shows high sequence similarity to mammalian YKT6, including conserved serine and threonine residues corresponding to the mammalian phosphorylation sites on Thr156, Ser181 and Ser174 (Fig. 1A). Similar to what is seen in yeast, *C. elegans* YKT-6 is essential for viability as *ykt-6* RNAi treatment throughout development leads to very slow growth, and deletion of the *ykt-6* gene leads to larval arrest (Maekawa et al., 2009). However, whether *C. elegans* YKT-6 plays a role in autophagy has not been assessed so far. To address this question, we applied *ykt-6* RNAi only post hatching, allowing embryonic development to proceed unaffected. Depletion of YKT-6 throughout the larval and adult stages resulted in viable adult animals, which could then be analyzed for autophagy function. To monitor autophagosome formation, we quantified the number of GFP–LGG-1 puncta, the *C. elegans* GABARAP ortholog, in the epidermis in wild-type control or *atg-7* RNAi- and *ykt-6* RNAi-fed worms (Jenzer et al., 2014; Meléndez et al., 2003; Zhang et al., 2015). Similar to what was seen with *atg-7* RNAi, *ykt-6* RNAi caused a strong reduction in GFP–LGG-1 puncta formation, indicating that autophagosome formation is blocked (Fig. 7A,B). In addition, GFP–LGG-1 puncta size was decreased upon *ykt-6* RNAi, further pointing to an impairment in autophagosome biogenesis (Fig. 7C). To confirm that the autophagic flux was affected, wild-type control RNAi- or *ykt-6* RNAi-fed animals were treated with the lysosomal fusion inhibitor chloroquine. Treatment in wild-type worms resulted in a strong accumulation of GFP–LGG-1 puncta, whereas in YKT-6-depleted animals only a few GFP–LGG-1 puncta accumulated (Fig. 7D,E). Together, these findings demonstrate that the YKT6 function in autophagosome formation is conserved in *C. elegans*.

**Fig. 8.**
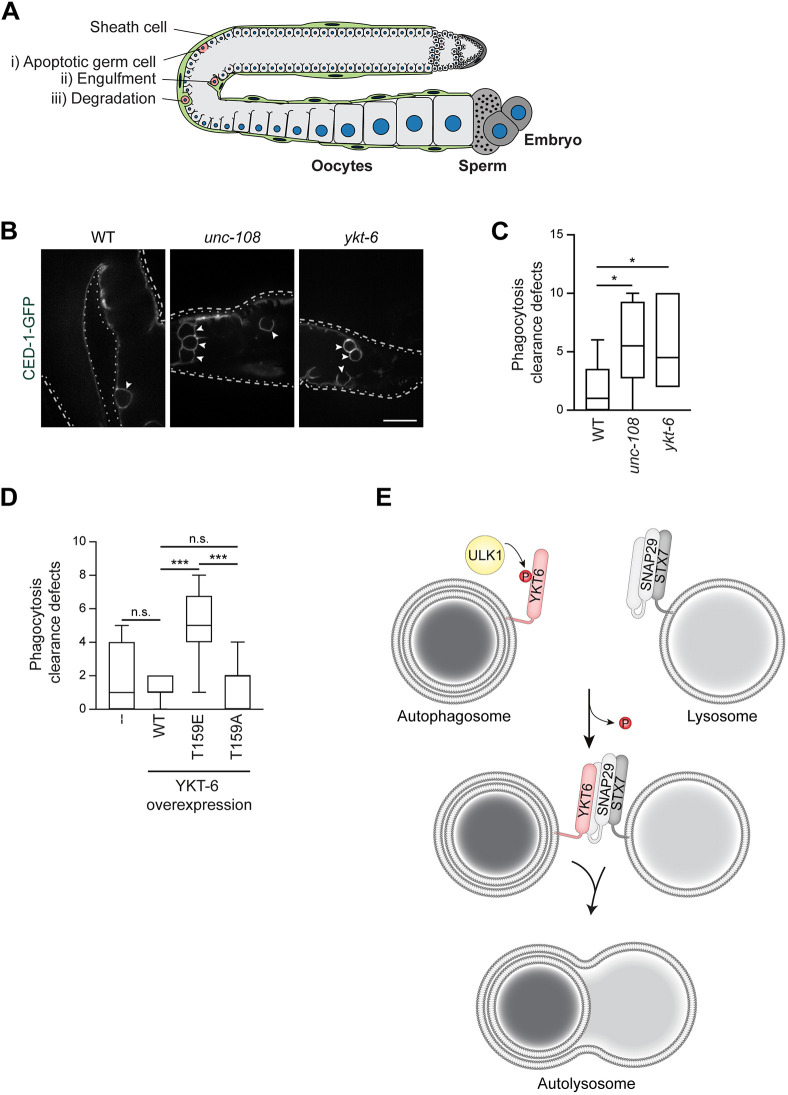
***C. elegans* YKT-6 is required for late steps in autophagy.** (A) Schematic representation of a *C. elegans* gonad arm (light gray), with the germ cell nuclei (blue) and spermatheca. Sheath cells (green) enwrap the gonad and scan the quality of the maturing germ cells. Dying germ cells undergo apoptosis (red, i), the resulting cell corpse is engulfed by the sheath cells by phagocytosis (ii), in which the corpse is removed by lysosomal degradation (iii). (B,C) In the sheath cells of *C. elegans* the indicated RNAi was performed. The accumulation of engulfed cell corpses labelled with CED-1–GFP (arrowheads) was visualized by fluorescent microscopy and analyzed in the gonads of 10 worms and represented in a box and whiskers plot (C). The box represents the 25–75th percentiles, and the median is indicated. The whiskers show the range. Dashed lines highlight the edge of the gonad arm. *P<0.05 (one-way ANOVA followed by a Bonferroni post-hoc test). Scale bar: 20 µm. Images are representative of three independent biological replicates. (D) The indicated YKT6 constructs were overexpressed selectively in sheath cells. The formation of phagosomes and their fusion with lysosomes was monitored by live-cell imaging (control: *n*=12, WT: *n*=21, T159E: *n*=7, T159A: *n*=14). The data is summarized in a box and whiskers plot, where the box represents the 25–75th percentiles, and the median is indicated. The whiskers show the range. ***P<0.001; n.s., not significant, *P*>0.05 (one-way ANOVA followed by a Bonferroni post-hoc test). (E) Model of YKT6 phospho-regulation during autophagy. ULK1 phosphorylates YKT6 located on autophagosomes to prevent their premature fusion with lysosomes. After dephosphorylation, YKT6 is able to form a complex with the SNARE proteins SNAP29 and STX7, allowing autophagosome–lysosome fusion.

As autophagosome formation is impaired after knockdown of *ykt-6*, it is difficult to assess the role of YKT-6 in autophagosome–lysosome fusion. We therefore turned to analyze YKT-6 function during LC3-associated phagocytosis (LAP) of apoptotic cells in the germline of *C. elegans* (Fig. 8A). Whereas phagosome formation happens independent of the autophagy machinery, the fusion of phagosomes with lysosomes requires many of the proteins that are also known to be involved in autophagosome–lysosome fusion (Huang et al., 2013; Jenzer et al., 2019; Li et al., 2012, 2013). The formation of phagosomes and their fusion with lysosomes can be monitored by following the GFP-tagged engulfment receptor CED-1, which is present in the sheath cells surrounding the gonad (Jenzer et al., 2019; Li et al., 2013; Zhou et al., 2001). CED-1 recognizes an ‘eat-me’ signal displayed on apoptotic cells. Thus, when dying germ cells are taken up by the adjacent sheath cells via phagocytosis, CED-1 starts clustering around the phagocytosed cell corpse. The formation of a closed CED-1–GFP ring therefore indicates that the engulfment is complete. Under normal conditions, this is then followed by the fusion of the phagosomes with lysosomes, leading to the shrinking and eventual disappearance of the phagosome (Jenzer et al., 2019; Li et al., 2013). To test whether YKT-6 is required for phagosome–lysosome fusion during LAP, *ykt-6* RNAi was performed only in the engulfing sheath cells (Fig. 8A), allowing the development of healthy worms. In wild-type control RNAi animals expressing CED-1–GFP, only a few engulfed cell corpses could be detected (Fig. 8B). In contrast, *ykt-6* RNAi led to an increase in the number of engulfed cell corpses remaining in the cytosol, similar to what is seen in *unc-108* (also known as *rab-2*) RNAi animals (Fig. 8B,C). The latter mutant is known to be defective in the efficient fusion of phagosomes with lysosomes, leading to impaired cell corpse degradation (Mangahas et al., 2008). To follow the dynamics of the engulfment and degradation process, we monitored the progression of phagocytotic removal of cell corpses in the germline by time-lapse live-cell microscopy. Both in wild-type control- and *ykt-6* RNAi-treated worms, phagosomes formed normally in sheath cells after ∼30 min. In control RNAi-treated worms, phagosomes fused with lysosomes within 50 min after formation (Fig. S4A). In contrast, upon *ykt-6* RNAi, this fusion was largely blocked, and phagosomes remained in the cytoplasm for the entire course of live-cell imaging of more than two hours (Fig. S4A).

To study whether YKT-6 phosphorylation also regulates phagosome–lysosome fusion during LAP in *C. elegans*, we mutated the conserved threonine 159 to non-phosphorylatable alanine or phospho-mimetic glutamate. These constructs were overexpressed specifically in the engulfing sheath cells of wild-type worms, and phagosome formation and subsequent fusion with lysosomes was monitored. Whereas the phagosome formation process was not affected, phagosome–lysosome fusion was inhibited by YKT-6 T159E overexpression, while fusion occurred normally in wild-type worms when wild-type YKT-6 or YKT-6 T159A were overexpressed (Fig. 8D; Fig. S4B). These findings indicate that YKT-6 phosphorylation also regulates phagosome–lysosome fusion in *C. elegans*.

Taken together, these findings suggest that *C. elegans* YKT-6, similar to yeast and mammalian YKT6, is required for both the formation of autophagosomes and their fusion with lysosomes, and that the fusion step is regulated by phosphorylation.

## DISCUSSION

Autophagosome fusion with lysosomes is promoted by two distinct SNARE complexes, one of them containing the evolutionarily conserved SNARE protein YKT6. In this study, we elucidated how autophagosome–lysosome fusion mediated by YKT6 is regulated in mammals. Previous reports in yeast have suggested that Ykt6 is recruited to autophagosome precursor membranes during autophagosome formation (Gao et al., 2020). There, Ykt6 is then kept inactive by Atg1-dependent phosphorylation, which prevents Ykt6 from bundling with the vacuolar SNARE proteins and hinders aberrant premature fusion of incomplete autophagosomes with the vacuole (Barz et al., 2020; Gao et al., 2020). Importantly, phosphorylation of the Ykt6 SNARE domain by Atg1 in yeast does not affect its general membrane association, but rather regulates its fusogenic activity on membranes. The SNARE domain of mammalian YKT6 has also been reported to be phosphorylated at Ser174, Ser181 and Thr187 (Karuna et al., 2020; Linnemannstöns et al., 2020; McGrath et al., 2021). However, in contrast to yeast Ykt6, these phosphorylation events result in conformational changes of YKT6 leading to an enhanced membrane binding (Karuna et al., 2020; Linnemannstöns et al., 2020; McGrath et al., 2021), and as a consequence to alterations in the secretory pathway, Ca^2+^ signaling and autophagy (Karuna et al., 2020; Linnemannstöns et al., 2020; McGrath et al., 2021). High-throughput screening identified phosphoinositide-dependent kinase 1 (PDPK1; also known as PDK1) and protein kinase Cι (PRKCI) as potential kinases that phosphorylate those sites in the SNARE domain of YKT6 (Karuna et al., 2020; McGrath et al., 2021). In this study, we define that the central autophagy kinase ULK1 specifically phosphorylates YKT6 on Thr156. Similar to what occurs in yeast, this inhibits the efficient bundling of YKT6 with the lysosomal SNARE protein SNAP29. Subsequently, autophagosome–lysosome fusion is blocked to prevent fusion of premature autophagosomes with lysosomes (Fig. 8E). It is noteworthy that neither phosphorylation of Thr156 nor the presence of ULK1 modify the association of YKT6 with the autophagosome or other membranes. Thus, the mechanism by which ULK1 phospho-regulates YKT6 function is substantially different to the phospho-regulation of YKT6 reported in earlier studies. Thr156 phosphorylation seems to control the local function of YKT6, rather than its spatial distribution, as observed for the previously described phospho-variants of YKT6.

Although the general mechanism of phospho-regulated SNARE bundling is conserved between yeast and mammals, there are some differences. In yeast, Atg1 directly phosphorylates Ser182 and/or Ser183 in the Ykt6 SNARE domain, whereas in mammals the prominent ULK1 target site is Thr156. The latter phosphorylation site is conserved in yeast Ykt6 (Thr158) and is also required for autophagy function in yeast cells. Although it has not been found to be a direct target of the Atg1 kinase *in vitro* (Barz et al., 2020), it is phosphorylated in an Atg1-dependent manner *in vivo* (Hu et al., 2019). Ser183 is also conserved in humans (Ser181); however, this site is not a prominent ULK1 target site in our *in vitro* experiments, which is in agreement with our data that it does not influence autophagy progression (Fig. S3A,B), but it might be involved in other pathways requiring YKT6 function (Karuna et al., 2020; Linnemannstöns et al., 2020). Moreover, whereas in yeast Ser182 and Ser183 do not alter Ykt6 membrane localization, Ser181 in mammals regulates YKT6 membrane association, similar to what is found for Ser174 (Fig. 3C,D). This underlines the notion that YKT6 regulation is highly complex and that membrane-binding properties are tightly regulated for the specific pathways YKT6 is involved in. We therefore suggest that YKT6 function in different pathways is spatio-temporally regulated by different kinases, affecting either its membrane association and/or its function on membranes. In autophagy, ULK1 is the main regulator of YKT6 acting on autophagosomal membranes.

A possible explanation for the differences between yeast and other organisms studied is that in yeast Ykt6 is the only autophagosomal SNARE involved in autophagosome–vacuole fusion, whereas mammals also possess STX17 on autophagosomes, which acts in a partially redundant manner. The different mechanisms of membrane binding, a lipid-anchor for YKT6 and a tandem transmembrane domain for STX17, could give them selectivity towards certain types of autophagosomes, with YKT6 targeting preferentially small, highly curved selective autophagosomes, whereas STX17 might bind to larger, less curved bulk autophagosomes (Kriegenburg et al., 2019). This notion agrees with the observation that STX17 is not required for mitophagy (Nguyen et al., 2016). In contrast, we found that the presence of a fusion-defective form of YKT6 impairs a proper mitophagy response, resulting in reduced cell survival (Fig. 6A–C). If YKT6 is also the main SNARE involved in other forms of selective autophagy, and how it cooperates with STX17, remains to be addressed.

Another question to be answered is the nature of the phosphatase reverting YKT6 phosphorylation in yeast and mammals. The role of a reversible phospho-switch bears similarities to the regulation of ATG4. ATG4 phosphorylation by ULK1 in mammals and Atg1 in yeast prevents the premature removal of LC3 proteins and Atg8, respectively, from the growing autophagosome (Pengo et al., 2017; Sánchez-Wandelmer et al., 2017). After autophagosome closure, ATG4 reactivation by dephosphorylation, likely by the PP2A phosphatase, allows the removal of LC3 from the outer autophagosomal membrane (Pengo et al., 2017). Therefore, similar to ATG4, YKT6 also needs to be kept inactive until later stages of autophagy to prevent premature fusion of immature autophagic membranes with the lytic compartment. The only phosphatase linked to YKT6 so far is calcineurin, which has been suggested to dephosphorylate YKT6 at Ser174 (McGrath et al., 2021). If the same phosphatase acts on YKT6 as on ATG4, or different phosphatases revert ULK1 phosphorylation on these target sites, remains yet to be understood.

In addition, we find that *C. elegans* YKT-6 is also required for autophagy function in worms and is regulated by conserved phosphorylation, highlighting the evolutionary importance of this SNARE protein in autophagy. Previously, the SNARE protein YKT6 has been identified to function in autophagosome–lytic compartment fusion in yeast, mammals and flies (Bas et al., 2018; Gao et al., 2018; Matsui et al., 2018; Takáts et al., 2018). Similar to yeast and mammalian YKT6, YKT-6 from *C. elegans* is also required for both early autophagosome formation and autophagosome–lysosome fusion. How and where *C. elegans* YKT-6 acts, and whether it plays a direct or rather a regulatory role, needs further investigation. It also remains to be investigated whether YKT-6 from *C. elegans* directly functions on autophagosomes and bundles with three Q-SNARES located on the lytic compartment, similar to yeast and mammals, or whether its function is more similar to *Drosophila melanogaster* Ykt6. DmYkt6 has been described to rather localize to lysosomal structures and to regulate trans-SNARE assembly (Takáts et al., 2018). Further studies on the localization and regulation of *C. elegans* YKT-6 are required to understand its spatio-temporal regulation.

## MATERIALS AND METHODS

### Plasmid construction

For the generation of HA–YKT6 plasmids, YKT6 from pMRXIP-Myc-YKT6 (Addgene #116945) was subcloned into a pcDNA3.1-HA backbone (Addgene #128034) using NotI. The resulting vector was used as a template to generate HA-YKT6 mutants using mutagenesis PCR. mScarlet–YKT6 plasmids were generated by replacing the HA sequence with mScarlet from pScarlet-N1 (Addgene #128060) using NheI and KpnI. The Halo-LC3 plasmid was generated by subcloning from pMRX-IP-HaloTag7-LC3 (Addgene #184899) into pIRESpuro3 (Clontech #631619) using NheI and NotI.

GST and the GST–Atg19 C-terminal fragment are described in Pfaffenwimmer et al. (2014). GST–YKT6 peptide plasmids were generated by restriction cloning of annealed 50-60mer complementary DNA fragments into pGEX4T1 (27458001, Amersham) via BamHI/XhoI digestion. The GST–YKT6 SNARE domain (amino acids 139–192 of human YKT6) was generated by restriction cloning of the PCR product into pGEX4T1 via BamHI/NotI digestion. Mutant variants were created by mutagenesis PCR.

For sheath cell-specific expression in *C. elegans* the *lim-7* promoter from plasmid pGC235 (Addgene #19683) was used. YKT-6 cDNA and T159E and T159A mutant versions were cloned as an AgeI/BssHII fragment and expressed unfused, while LAAT-1 cDNA was C-terminally fused to mCherry.

Other plasmids used in this study were pEGFP-hLC3 (Addgene #87872), mCherry-LC3 (Addgene #40827) and tfLC3 (Addgene #21074).

### Protein expression

GST and GST fusion proteins were expressed in BL21(DE3) cells (69450, Novagen). After IPTG induction, cells were grown at 16°C for 16 h or at 37°C for 3 h. For the isolation of GST and GST fusion proteins, harvested cells were resuspended in lysis buffer [phosphate-buffered saline (PBS), 10% glycerol, 1% Triton X-100 supplemented with cOmplete™ protease inhibitor cocktail (EDTA-free, Roche) and 1 mM PMSF] and lysed by sonication. Debris were removed by centrifugation at 20,000 ***g*** for 10 min at 4°C, and the supernatant was incubated for 1 h at 4°C with GSH Sepharose^®^4B. After three washing steps with lysis buffer and two washing steps with PBS, the GST fusion proteins either remained bound to the GSH Sepharose for subsequent pulldown experiments or were eluted using 50 mM Tris-HCl pH 8.0 with 20 mM reduced GSH. Eluted proteins were rebuffered into PBS and concentrated using Amicon^®^ Ultra Centrifugal Filter devices and used in *in vitro* kinase assays.

### Pulldown experiments

To assess direct interaction of SNAP29 with the SNARE domain of YKT6, pulldown experiments were performed using immobilized GST–Ykt6 SNARE domains purified from *Escherichia coli* and incubated with cell extract from HEK293T cells (R78007, Thermo Fisher Scientific) containing endogenous SNAP29. Cell extract was obtained by lysing HEK293T cells in RLB+ buffer [1× PBS pH 7.4, 10% glycerol, 0.5% Tween-20, 1 mM sodium fluoride, 20 mM β-glycerophosphate, 1 mM PMSF, 1 mM sodium vanadate and cOmplete™ protease inhibitor cocktail (EDTA-free; Roche)] and brief sonication in a water bath and clearing at 2500 ***g*** for 2.5 min, 4°C. Similar amounts of immobilized GST fusion constructs were incubated with 2 mg precleared cell extracts from HEK293T cells for 1 h rotating end-over-end at 4°C. Beads were washed three times in RLB+ and once in PBS before elution in urea loading buffer (58 mM Tris-HCl pH 6.8, 2.45% glycerol, 4 M urea and 71.5 mM β-mercaptoethanol) and were subjected to SDS-PAGE followed by western blotting. Precipitated SNAP29 was detected using an anti-SNAP29 antibody. The amounts of GST fusion constructs were controlled by Ponceau staining.

### Antibodies

The following antibodies were used in this study: anti-LC3 (#3868, Cell Signaling Technology), anti-YKT6 (sc-365732, Santa Cruz Biotechnology), anti-vinculin (700062, Thermo Fisher Scientific), anti-p62 (H00008878-M01, Abnova), anti-SNAP29 (ab181151, Abcam), anti-NDP52 (ab68588, Abcam), anti-OPTN (HPA003360, Sigma Aldrich), anti-TOM20 (sc-17764, Santa Cruz Biotechnology), anti-HaloTag (G9211, Promega), anti-ULK1 (#8054, Cell Signaling Technology), anti-FIP200 (#12436, Cell Signaling Technology). All antibodies were used at a 1:1000 dilution for western blotting and 1:200 for fluorescence microscopy. Uncropped images of western blots are shown in Fig. S5.

### *In vitro* kinase assay

ULK1 purified from the FreeStyle™ 293-F cell line, a fast-growing variant of HEK293 cells (SRP0252, Sigma-Aldrich) was mixed with 3 µg of the indicated GST fusion constructs and 2 µCi γ-[^32^P]-ATP and incubated for 30 min at 30°C in 20 mM HEPES pH 7.4, 150 mM potassium acetate, 10 mM magnesium acetate, 0.5 mM EGTA, 5 mM NaCl and 10 mM Na_3_VO_4_. The reaction was stopped by the addition of urea loading buffer and the samples were analyzed by SDS-PAGE followed by phospho-imaging.

### Cell culture

HEK293 Flp-In T-REx (R78007, Thermo Fisher Scientific) and U2OS Flp-In T-REx cells (K650001, Thermo Fisher Scientific) were grown at 37°C in a humidified 5% (v/v) CO_2_-air atmosphere in Dulbecco's modified Eagle's medium (DMEM; D6429, Sigma Aldrich) containing 10% fetal bovine serum (FBS), 5 U/ml penicillin and 50 µg/ml streptomycin. To induce autophagy, cells were washed twice with PBS and the cells were grown for the indicated times in Earle's balanced salt solution (E3024, Sigma-Aldrich) with 300 nM Torin 1 (#14379, Cell Signaling Technology).

Unless otherwise indicated, all cells were treated with a cocktail of two siRNA to remove endogenous expression of YKT6 (YKT6 siRNA1: 5′-AAGUUCCUGCGAGAAAUGGAUUU-3′; YKT6 siRNA2: 5′-AAGUGAGAUCUCCGCUUGCAGUU-3′). siRNAs were transfected using ViaFect (E4981, Promega) according to the manufacturer's instructions.

When indicated, cells were treated with 200 nM Bafilomycin A1 (54645, Cell Signaling Technology), 200 nM wortmannin (W1628, Sigma-Aldrich), 200 nM TMR HaloTag ligand (G8251, Promega) or 10 µM antimycin A (A8674, Sigma-Aldrich) and 10 µM oligomycin (75351, Sigma-Aldrich) for the indicated times.

All cells were tested for mycoplasm contamination before experiments.

### Fluorescence microscopy of mammalian tissue culture cells

For fixed samples, cells grown on glass coverslips were fixed with 4% paraformaldehyde in PBS for 10 min, permeabilized with 0.1% Triton X-100 in PBS for 30 min, blocked with 5% BSA in PBS for 45 min, and then incubated overnight with primary antibodies diluted 1:200 in 1% BSA in PBS. After washing, cells were incubated with Alexa Fluor cross-adsorbed secondary antibodies (Invitrogen) at a 1:1000 dilution for 60 min.

For live-cell imaging, cells were grown in 35 mm glass base dishes (Ibidi), permeabilized with 50 µg/ml of digitonin (Matrix BioScience) in PBS for 15 min and preserved in an environmental chamber at 37°C, 5% CO_2_ during image acquisition.

HEK293T cells were imaged using a DeltaVision Ultra High Resolution Microscope with UPlanSApo 100×/1.4 oil Olympus objective, using a sCMOS pro.edge camera at room temperature (GE Healthcare, Applied Precision). U2OS cells were imaged using a DeltaVision OMX Flex Microscope with UPlanSApo 60×/1.4 oil Olympus objective, using a PCO Edge 4.2 sCMOS camera.

### *In vitro* fusion assay

Autophagosomes were enriched from 48 h YKT6 siRNA-treated HEK293T cells stably expressing mCherry–LC3 and the indicated HA–YKT6 constructs after 3 h treatment with 300 nM Torin 1. Cells were collected in PBS by centrifugation at 500 ***g*** for 5 min and resuspended in 50 mM KCl, 100 mM KH_2_PO_4_ and 100 mM K_2_HPO_4_ with cOmplete™ protease inhibitor cocktail (EDTA-free, Roche). Cells were then lysed by 30 strokes in a glass douncer. After two preclearing steps (2000 ***g***, 5 min), the membrane fraction was pelleted by centrifugation at 20,000 ***g*** for 30 min and resuspended in cleared cytosol (see below). Lysosomes were obtained from HEK293T FIP200 KO cells stained with LysoTracker Green (L7526, Thermo Fisher Scientific), after 18 h treatment with 300 nM Torin 1. As FIP200 KO cells cannot form autophagosomes, the LysoTracker Green-positive structures isolated from these cells are lysosomes only, not autolysosomes. The membrane fraction was then prepared as described for autophagosomes. The cytosol was obtained from HEK293T WT cells. Cells were lysed as above, and the cytosolic supernatant was prepared by centrifugation at 20,000 ***g*** for 30 min.

The autophagosomal, lysosomal and cytosolic fractions were then supplemented with an ATP regeneration system (200 mM phosphocreatine, 0.5 mg/ml creatine kinase, 3 mM ATP and 0.3 mM GTP). Fusion reactions were incubated for 1 h at 37°C before mixing in a 1:1 ratio with low-melting agarose and mounted in glass slides for fluorescent microscopy visualization. Fusion was assessed by colocalization of mCherry–LC3 and LysoTracker Green, measured by Manders’ colocalization coefficient using Fiji (ImageJ). As a negative control, the membrane fractions were mixed in the absence of cytosol and the ATP regeneration system and imaged immediately.

### Live-cell imaging microscopy of *C. elegans*

Mounting of adult worms has been described in Gomes et al. (2016). Microscopy was performed with a VisiScope spinning disk confocal microscope system (Visitron Systems, Puchheim, Germany) based on a Leica DMi8 inverted microscope, a Yokogawa CSU X1 scan head, a Hamamatsu ORCA-Flash 4.0 CC1140 and a SuperResolution upgrade extension GATACA LiveSR system. All acquisitions were performed at 21–23°C, using a Leica HC PL APO 63×/1.4–0.6 oil objective or a Nikon spinning disc system equipped with a Yokogawa CSU-W1 scan head, Andor Xyla 4.2 sCMOS camera and PL Apo 60×/1.2 water objective.

The quantification of the number and size of LGG-1 puncta was done in the epidermis, as autophagosomes are well visible in these cells. Analysis was performed as described in Papandreou and Tavernarakis (2017). For the quantification of the size of LGG-1 puncta, threshold and intensity density measurements on Fiji (ImageJ) were used (Schindelin et al., 2012). For each image background correction was applied individually.

The phagocytosis of apoptotic cells in the *C. elegans* germline by the surrounding sheath cells was analyzed by using the *bcIs39* marker strain expressing the engulfment receptor CED-1–GFP fusion protein in the engulfing sheath cells. To count the number of phagosomes, threshold and quantification were performed using Fiji (ImageJ) (Yen et al., 1995). To analyze whether YKT-6 threonine 159 mutation affects germline phagocytosis of apoptotic cells, wild-type YKT-6, YKT-6 T159E or YKT-6 T159A was overexpressed specifically in the engulfing sheath cells under the control of the lim-7 promoter in transgenic wild-type worms expressing CED-1–GFP and the lysosomal membrane protein LAAT-1–mCherry (Liu et al., 2012). After phagosome closure, LAAT-1–mCherry positive lysosomes were tethered to and accumulate around the phagosome, allowing the monitoring of the exact timing of fusion between phagosomes and lysosomes. Phagosome formation and fusion with lysosomes was monitored in 1 day adult animals by spinning disc confocal live-cell imaging for up to 3 h with image acquisition every 2 min. Worms were immobilized on a 2% agarose pad with 300 µM levamisole.

### Proteinase protection assay

Cells treated for 2 h with EBSS and 300 nM Torin 1 were washed with PBS and collected by centrifugation at 500 ***g*** for 5 min. Pellets were resuspended in homogenization buffer [250 mM sucrose, 20 mM HEPES-KOH pH 7.4, 1 mM EDTA and cOmplete™ protease inhibitor cocktail (EDTA-free, Roche)] and lysed by 30 passages with a 25 G needle. After two preclearing steps (2000 ***g***, 5 min), cell membranes were pelleted by centrifugation at 20,000 ***g*** for 30 min. The pellet was resuspended in 100 µl of homogenization buffer without EDTA or protease inhibitors, divided into three equal fractions and incubated in the presence or absence of proteinase K (100 µg per ml of sample) with or without 0.5% Triton X-100 for 30 min on ice. The samples were then subjected to trichloroacetic acid (TCA) precipitation and resuspended in sample buffer.

### Immunoprecipitation

Cells were washed with PBS and lysed in TNE buffer (50 mM Tris-HCl pH 7.5, 150 mM NaCl, 1 mM EDTA) containing 1% Triton X-100 and protease inhibitors. The lysates were centrifugated at 15,000 ***g*** for 5 min and supernatants adjusted to the same protein concentration. Immunoprecipitation was conducted by incubation with 15 µl of HA–agarose beads (A2095, Sigma Aldrich) for 2 h at 4°C. After that, the beads were washed twice with TNE buffer containing 0.2% NP-40 and bound proteins were eluted with Laemmli SDS loading (LDS) buffer (62.5 mM Tris-HCl pH 6.8, 2% SDS, 10% glycerol, 0.1% Bromophenol Blue and 0.3 M β-mercaptoethanol) at 95°C. For LC-MS/MS analysis, instead of protein elution in LDS buffer, the beads were washed one more time without detergents and eluted with 2% sodium deoxycholate, 50 mM Tris-HCl pH 7.5 at 95°C.

### Membrane fractionation

Cells treated for 2 h with EBSS and 300 nM Torin 1 were washed with PBS and collected by centrifugation at 500 ***g*** for 5 min. Pellets were resuspended in PBS with cOmplete™ protease inhibitor cocktail (EDTA-free, Roche) and lysed by 30 passages with a 25 G needle. Following two preclearing steps (2000 ***g***, 5 min), cell membranes were pelleted by ultracentrifugation with an Optima MAX-130K Ultracentrifuge at 100,000 ***g*** for 1 h at 4°C. After separation of the cytosolic fraction, the membrane pellet was resuspended in PBS with 1% Triton X-100. All samples were mixed with LDS buffer and boiled for 5 min at 95°C before SDS-PAGE analysis.

### LDH sequestration assay

Cells treated for 4 h with EBSS, 300 nM Torin 1 and 200 nM Bafilomycin A1 were washed with PBS and collected by centrifugation at 500 ***g*** for 5 min. Pellets were resuspended in homogenization buffer [250 mM sucrose, 20 mM HEPES-KOH pH 7.4, 1 mM EDTA and cOmplete™ protease inhibitor cocktail (EDTA-free, Roche)] and lysed by 30 passages with a 25 G needle. After two preclearing steps (2000 ***g***, 5 min), cell membranes were pelleted by centrifugation at 20,000 ***g*** for 30 min. The membrane pellet was resuspended in 90 µl of LDH Assay Buffer (kit, MAK066, Merck) with 0.5% Triton X-100. The LDH activity of 5 µl of crude extract or membrane fraction was measured using the Lactate Dehydrogenase Activity Assay Kit (MAK066, Merck) with a Spark multimode microplate reader (Tecan). The relative activity was calculated as the ratio between membrane sequestered vs total LDH activity.

### MTT cell viability assay

5×10^4^ HEK293T cells were seeded in 96 well plates and subjected to 10 µM antimycin A and oligomycin treatment for 72 h. Afterwards, the cells were treated with the 3-(4,5-dimethylthiazol-2-yl)-2,5-diphenyltetrazolium bromide (MTT) Cell Proliferation Kit I (11465007001, Roche) following the manufacturer's instructions. The resulting colored product absorbance was measured at 560 nm.

### Worm strains and maintenance

Strains used in this study were cultivated on NGM (Nematode Growth Medium) agar plates at 20°C feeding on OP50 if not otherwise stated (Brenner, 1974). The following strains have been used: wild type Bristol N2, adIs2122[lgg-1p::GFP-LGG-1; rol-6(su1006)], bcIs39[lim-7p::ced-1::GFP; lin-15(+)], rde-4(ne301); bcIs39; gzEx777[lim-7p::rde-4cDNA; ttx-3::RFP], *bcIs39*; *gzEx752*[*lim-7p::LAAT-1-mCherry*; *rol-6(su1006)*], *bcIs39*; *gzEx782*[*lim-7p::YKT-6*; *lim-7p::LAAT-1-mCherry*; *rol-6(su1006)*], *bcIs39*; *gzEx786*[*lim-7p::YKT-6(T159E)*; *lim-7p::LAAT-1-mCherry*; *rol-6(su1006)*] and *bcIs39*; *gzEx788*[*lim-7p::YKT-6(T159A)*; *lim-7p::LAAT-1-mCherry*; *rol-6(su1006)*].

### RNA interference in *C. elegans*

RNAi knockdown experiments were performed by feeding worms HT115(DE3) bacteria expressing double stranded RNA (dsRNA) for each gene of interest according to the standard protocol (Hammell and Hannon, 2012). To allow the embryonic development to proceed undisturbed, worms were exposed to the double dsRNA from hatching to adulthood. The RNAi phenotypes were analyzed in 1-day-old adult animals. The cDNAs of the genes of interest were cloned into the standard RNAi vector L4440 by restriction cloning, confirmed by sequencing and transformed into HT115(DE3) bacteria. Control worms were fed bacteria containing the empty vector L4440. To deplete YKT-6 selectively in the sheath cells we used an RNAi deficient *rde-4* strain which was rescued by expressing the *rde-4* cDNA exclusively in the sheath cells under control of the *lim-7* promoter from an extra-chromosomal array (Sasidharan et al., 2012).

### Sample preparation for LC-MS/MS

Samples eluted from immunoprecipitation experiments were reduced and alkylated by adding 1 mM TCEP, 4 mM chloroacetamide (final concentration) in 50 mM Tris-HCl pH 8.5 and incubation for 10 min at 95°C. Digestion was performed with 500 ng LysC (Wako Chemicals, 125-02543) and 500 ng Trypsin (Promega, V5113) in 50 mM Tris-HCl pH 8.5 overnight (16 h) at 37°C and cleaned-up according to the iST protocol (Kulak et al., 2014). Briefly, digestion was stopped with the same volume of 1% trifluoroacetic acid (TFA) in isopropanol and directly loaded on in-house assembled SDB-RPS STAGE tips. Following two wash steps with 1% TFA in isopropanol and 0.2% TFA in water, peptides were eluted with 1.25% ammonium hydroxide in 80% ACN and dried for storage at −20° until LC-MS/MS measurements.

### LC-MS/MS analyses

Samples were analyzed on a Q Exactive HF coupled to an easy nLC 1200 (ThermoFisher Scientific) using a 35 cm long, 75 µm ID home-made fused-silica emitter packed with 1.9 µm C18 particles (Reprosil pur, Dr. Maisch), and kept at 50°C using an integrated column oven (Sonation). Peptides were eluted by a linear gradient from 4-32% acetonitrile over 60 min and directly sprayed into the mass-spectrometer equipped with a nanoFlex ion source (Thermo Fisher Scientific). Full scan MS spectra (350–1650 m/z) were acquired in Profile mode at a resolution of 60,000 at *m*/*z* 200, a maximum injection time of 20 ms and an AGC target value of 3×10^6^ charges. Up to 10 peptides per full scan were isolated using a 1.4 Th window and fragmented using higher energy collisional dissociation (normalized collision energy of 27). MS/MS spectra were acquired in centroid mode with a resolution of 30,000, a maximum injection time of 54 ms and an AGC target value of 10^5^. Singly charged ions, ions with a charge state above 5 and ions with unassigned charge states were not considered for fragmentation and dynamic exclusion was set to 20 s to minimize selection of already fragmented precursors.

### Mass spectrometry data processing

MS raw data processing was performed with MaxQuant (v 1.6.17.0) (Tyanova et al., 2016b). Acquired spectra were searched against the human reference proteome protein sequences (Taxonomy ID 9606) downloaded from UniProt (“One Sequence Per Gene”; 17-Apr-2022; 20509 sequences without isoforms) and sequences of the variants of YKT6 as well as a collection of common contaminants (244 entries) using the Andromeda search engine integrated in MaxQuant (Cox et al., 2011). Identifications were filtered to obtain false discovery rates (FDR) below 1% for both peptide spectrum matches (PSM; minimum length of 7 amino acids) and proteins using a target-decoy strategy (Elias and Gygi, 2007).

### MS statistical data analysis and visualization

First, proteins identified by a single modified peptide, reversed proteins from the decoy database and contaminant proteins (Proteases, Keratins) were removed from ‘proteinGroups.txt’. In order to obtain a ‘common’ YKT6 abundance without losing quantitative information, intensities from the expected YKT6 variants were added to the intensity of the WT for each sample, because the protein grouping algorithm from MaxQuant always allocated the shared peptides to the WT due to principles of parsimony. Only proteins quantified in all three replicates in at least one group were considered for further analysis. iBAQ intensities from MaxQuant were normalized by global intensity (GI), normalization and statistical analysis were performed with limma, both using the NormalyzerDE package (Willforss et al., 2019). GI-normalized intensities were adjusted to YKT6 abundance using the ‘Subtract row cluster’ function in Perseus (v. 1.6.15.0) before statistical analysis (Tyanova et al., 2016a).

### Statistics

Data from western blotting, LDH activity and the survival assay is represented in bar graphs displaying the mean of each biological replicate ±s.e.m. Microscopy quantifications are represented using box and whisker plots, with whiskers ranging from the minimum to maximum values, and dots indicating the mean of each biological replicate. At least three independent biological replicates were performed for each experiment. When two samples were compared, a two-tailed unpaired Student's *t*-test was employed. To compare three or more samples a one-way ANOVA followed by a Bonferroni post-hoc test was applied to allow multiple comparisons. At least three independent biological replicates were acquired for each shown experiment. Statistical details can be found in the figure legends for each experiment. The level of significance is shown in asterisks as follows: ***P<0.001, **P<0.01, *P<0.05, not significant (n.s.) *P*>0.05.

## Supplementary Material

10.1242/joces.260546_sup1 Supplementary information
