# Supplementary material for: ULK1-mediated phosphorylation regulates the conserved role of YKT6 in autophagy
Source: J Cell Sci. 2023 Feb 10;136(3):jcs260546. doi: 10.1242/jcs.260546 (PMC10022743; doi:10.1242/jcs.260546)
Supplement: Supplementary information [file joces-136-260546-s1.pdf]

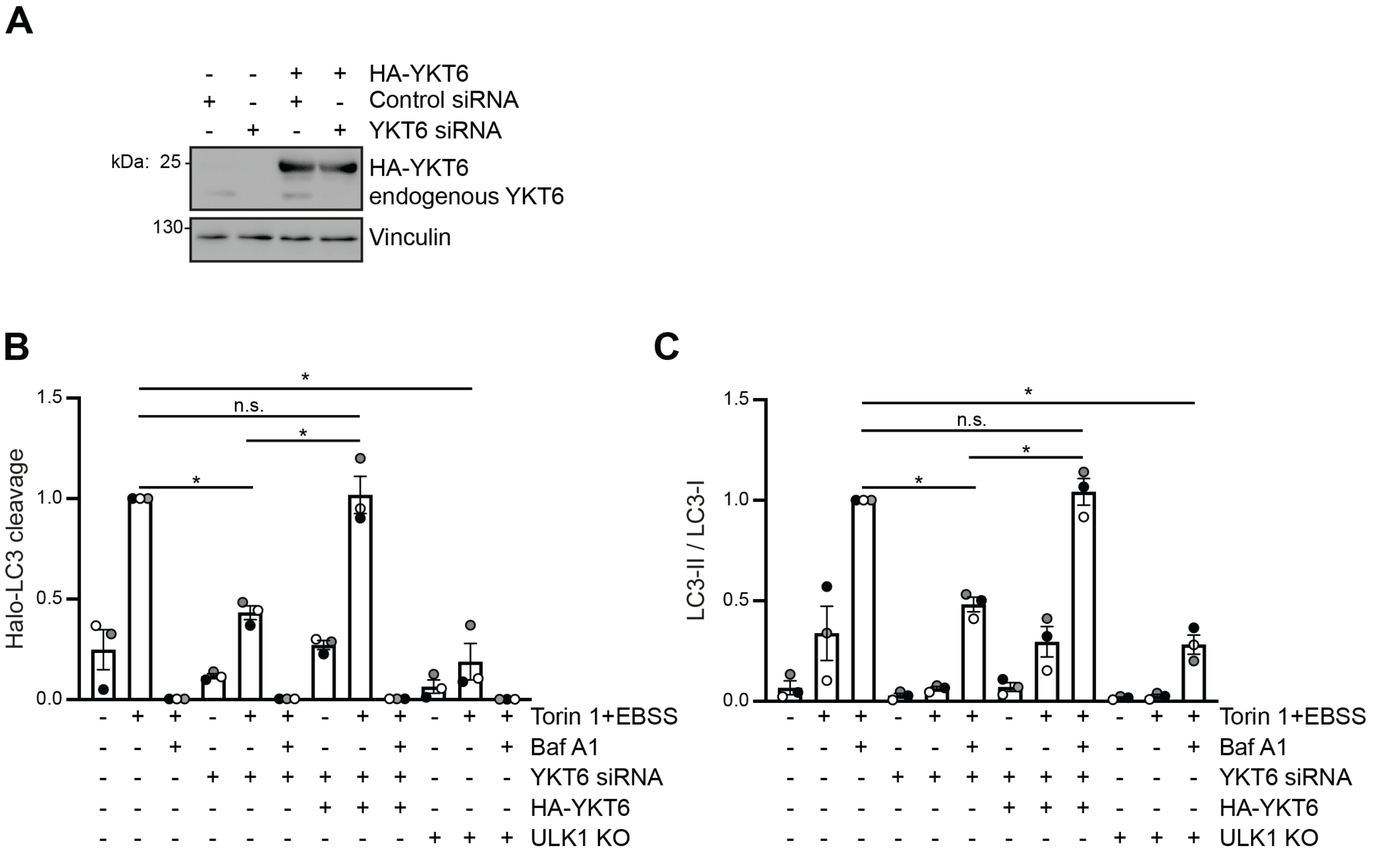

Fig. S1. Autophagy defects after depletion of *YKT6* are rescued by exogenous YKT6 expression.

A) HEK293T WT cells stably expressing siRNA-resistant HA-YKT6-WT as indicated were transfected with control siRNA or YKT6 siRNA for 48 h. Samples were analyzed by Western blotting using anti-YKT6 antibodies. Images are representative of three independent biological replicates.

B-C) HaloTag cleavage and LC3 lipidation from Fig 1D were analyzed by Western blotting. Densitometric quantification of cleaved HaloTag relative to Vinculin (B) or the LC3-II to LC3-I ratio relative to Vinculin (C) was performed and is shown as means ± SEM, with dots indicating the value of each biological replicate. Statistical analysis was performed using a one-way ANOVA followed by a Bonferroni post-hoc test, *$p < 0.05$, not significant (n.s.) $p > 0.05$.

Journal of Cell Science • Supplementary information

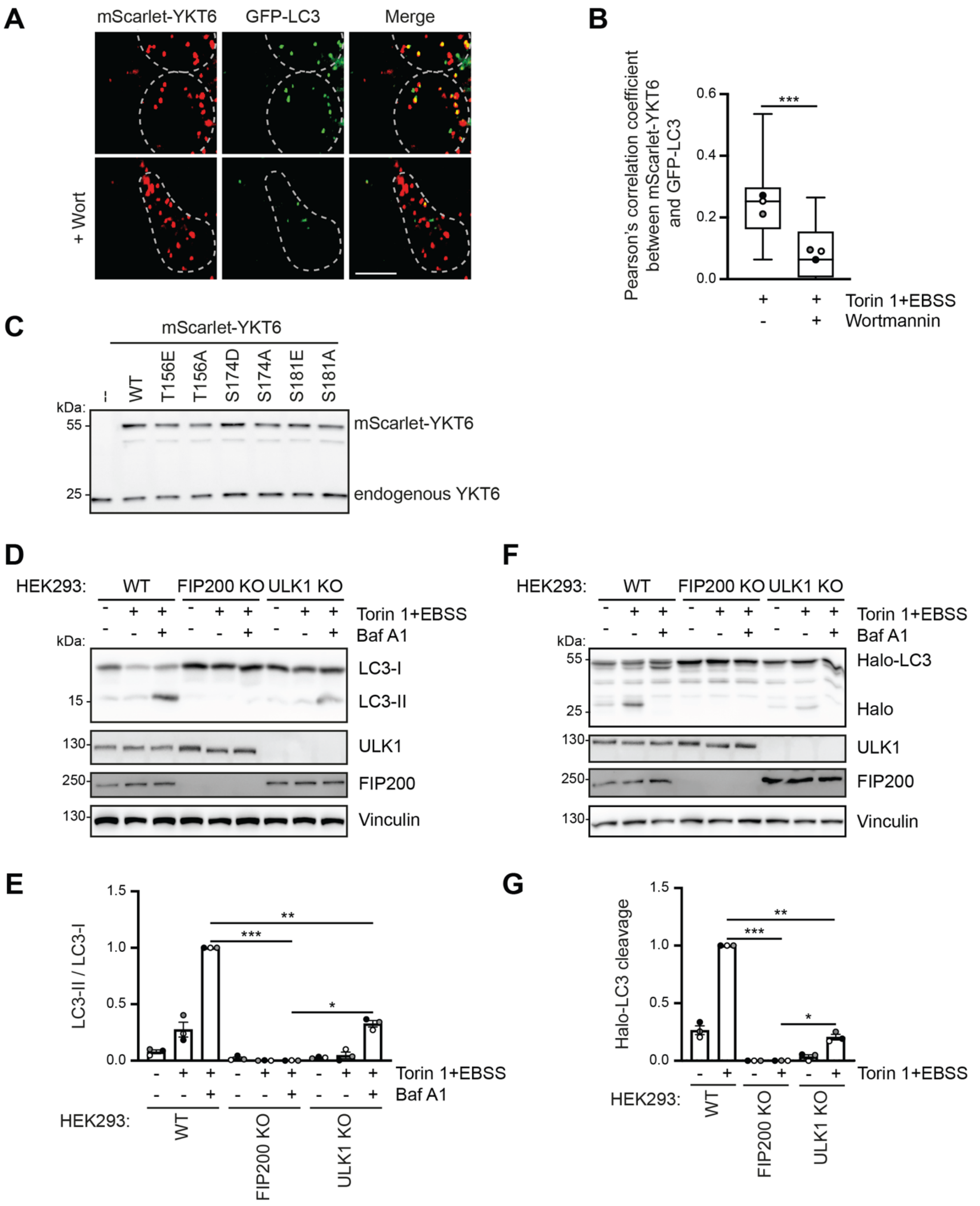

Fig. S2. Validation of control cell lines

A and B) YKT6 siRNA treated HEK293T cells stably expressing GFP-LC3 were transiently transfected with mScarlet-YKT6-WT for 24 h and treated for 4 h with EBSS and 300 nM Torin 1 as well as 200 nM Wortmannin as indicated. After 15 min permeabilization with 50 μg/ml of digitonin, cells were visualized by fluorescence microscopy. Colocalization of mScarlet-YKT6 and GFP-LC3 was quantified in >30 cells and represented in a box and whiskers plot, with dots

Journal of Cell Science • Supplementary information

indicating the mean of each biological replicate (B). Statistical analysis was performed using a *t*-test assuming unequal variances, ***$p < 0.001$. Scale bar: 5 µm. Images are representative of three independent biological replicates.

C) Expression levels of mScarlet-YKT6 variants used in Fig 3C were analyzed by Western blotting. Images are representative of three independent biological replicates.

D and E) HEK293T WT, FIP200 KO or ULK1 KO cells were grown for 4 h either in DMEM or EBSS with 300 nM Torin 1 with or without 200 nM Bafilomycin A1 as indicated. LC3 lipidation was analyzed by Western blotting (D). Densitometric quantification of the LC3-II to LC3-I ratio relative to Vinculin was performed and is shown as means ± SEM, with dots indicating the value of each biological replicate (E). Statistical analysis was performed using a one-way ANOVA followed by a Bonferroni post-hoc test, *$p < 0.05$, **$p < 0.01$, ***$p < 0.001$. One out of three independent biological replicates is shown.

F and G) Cells from D) stably expressing Halo-LC3 were treated with 200 nM TMR HaloTag ligand for 10 min. The ligand was removed by washing two times with PBS. Subsequently, the cells were grown for 4 h either in DMEM or EBSS with 300 nM Torin 1 with or without 200 nM Bafilomycin A as indicated. Densitometric quantification of cleaved HaloTag relative to Vinculin was performed and is shown as means ± SEM, with dots indicating the value of each biological replicate (G). Statistical analysis was performed using a one-way ANOVA followed by a Bonferroni post-hoc test, *$p < 0.05$, **$p < 0.01$, ***$p < 0.001$. One out of three independent biological replicates is shown.

Journal of Cell Science • Supplementary information

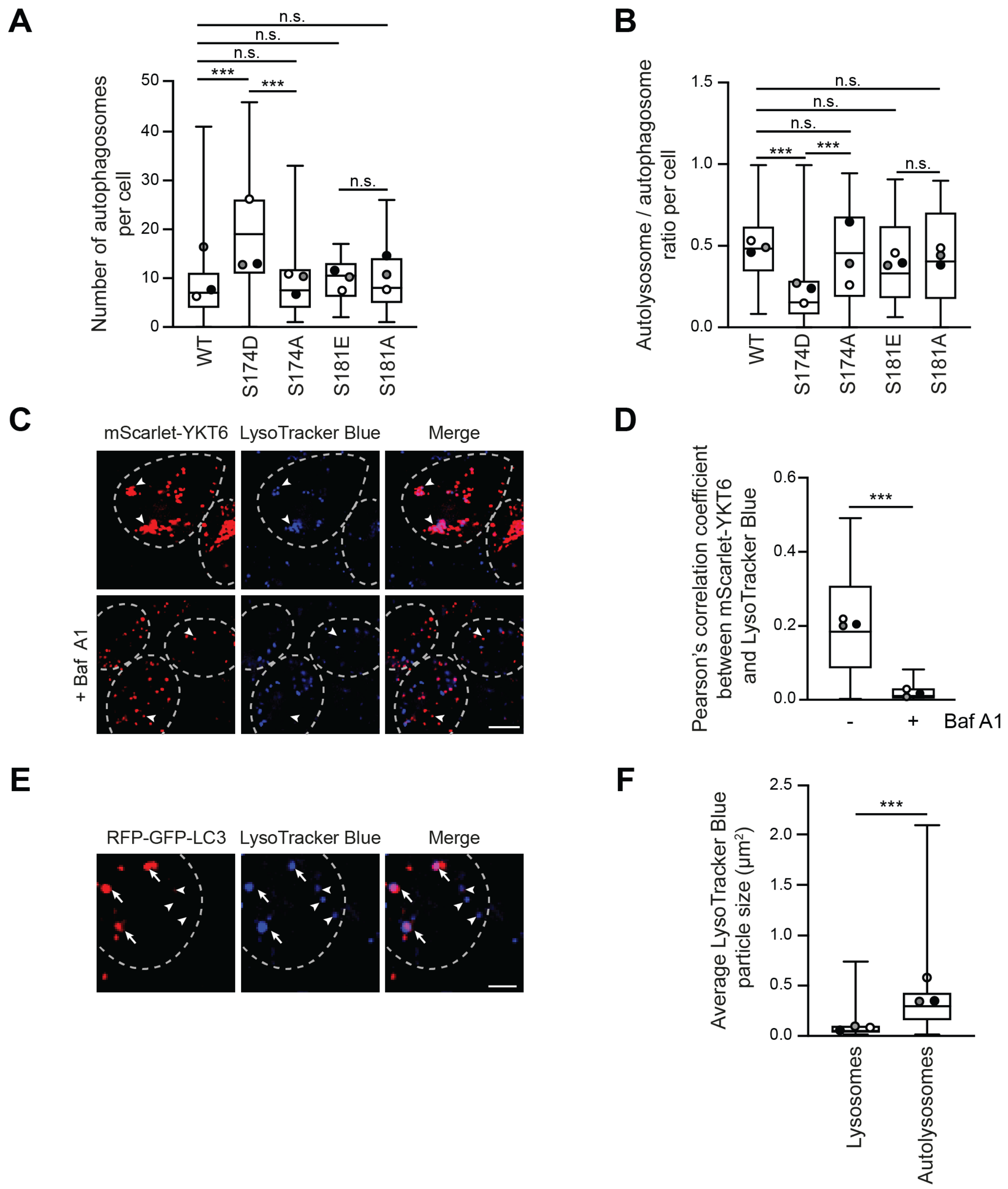

Fig. S3. Additional YKT6 phospho-mutants in autophagy

A and B) YKT6 siRNA treated HEK293T cells stably expressing the indicated HA-YKT6 constructs were transiently transfected with RFP-GFP-LC3 for 24 h and treated for 4 h with EBSS and 300 nM Torin 1. After 15 min permeabilization with 50 µg/ml of digitonin, cells were visualized by fluorescence microscopy. Autophagosomes (green and red puncta) and autolysosomes (red-only puncta) were quantified from > 30 cells. The number of autophagosomes (A) and the ratio of autolysosomes to autophagosomes (B) is shown in a box and whiskers plot, with dots indicating the mean of each biological replicate. Statistical analysis was performed using a one-way ANOVA followed by a Bonferroni post-hoc test, $^{**}p < 0.01$, $^{***}p < 0.001$, not significant (n.s.) $p > 0.05$. Three independent biological replicates were performed.

Journal of Cell Science • Supplementary information

C and D) YKT6 siRNA treated HEK293T cells transiently expressing mScarlet-YKT6 were treated for 4 h with EBSS and 300 nM Torin 1 as well 200 nM Bafilomycin A1 as indicated. After 3 h, 100 nM LysoTracker Blue was added for 45 min, followed by 15 min permeabilization with 50 µg/ml digitonin. Cells were then visualized by fluorescence microscopy. The colocalization of mScarlet-YKT6 and LysoTracker Blue puncta was quantified from >30 cells and represented in a box and whiskers plot, with dots indicating the mean of each biological replicate (D). Statistical analysis was performed using a *t*-test assuming unequal variances, ***$p < 0.001$. Scale bar: 5 µm. Images are representative of three independent biological replicates.

E and F) HEK293T cells stably expressing RFP-GFP-LC3 were treated for 4 h with EBSS and 300 nM Torin 1 and for the last 45 min with 100 nM LysoTracker Blue. After 15 min permeabilization with 50 µg/ml of digitonin cells were visualized by fluorescence microscopy (only the red channel is shown for RFP-GFP-LC3). Arrowheads: lysosomes, arrows: autolysosomes. The particle size of LysoTracker Blue puncta was quantified from >30 cells and represented in a box and whiskers plot, with dots indicating the mean of each biological replicate (F). Statistical analysis was performed using a *t*-test assuming unequal variances, ***$p < 0.001$. Scale bar: 2 µm. Images are representative of three independent biological replicates.

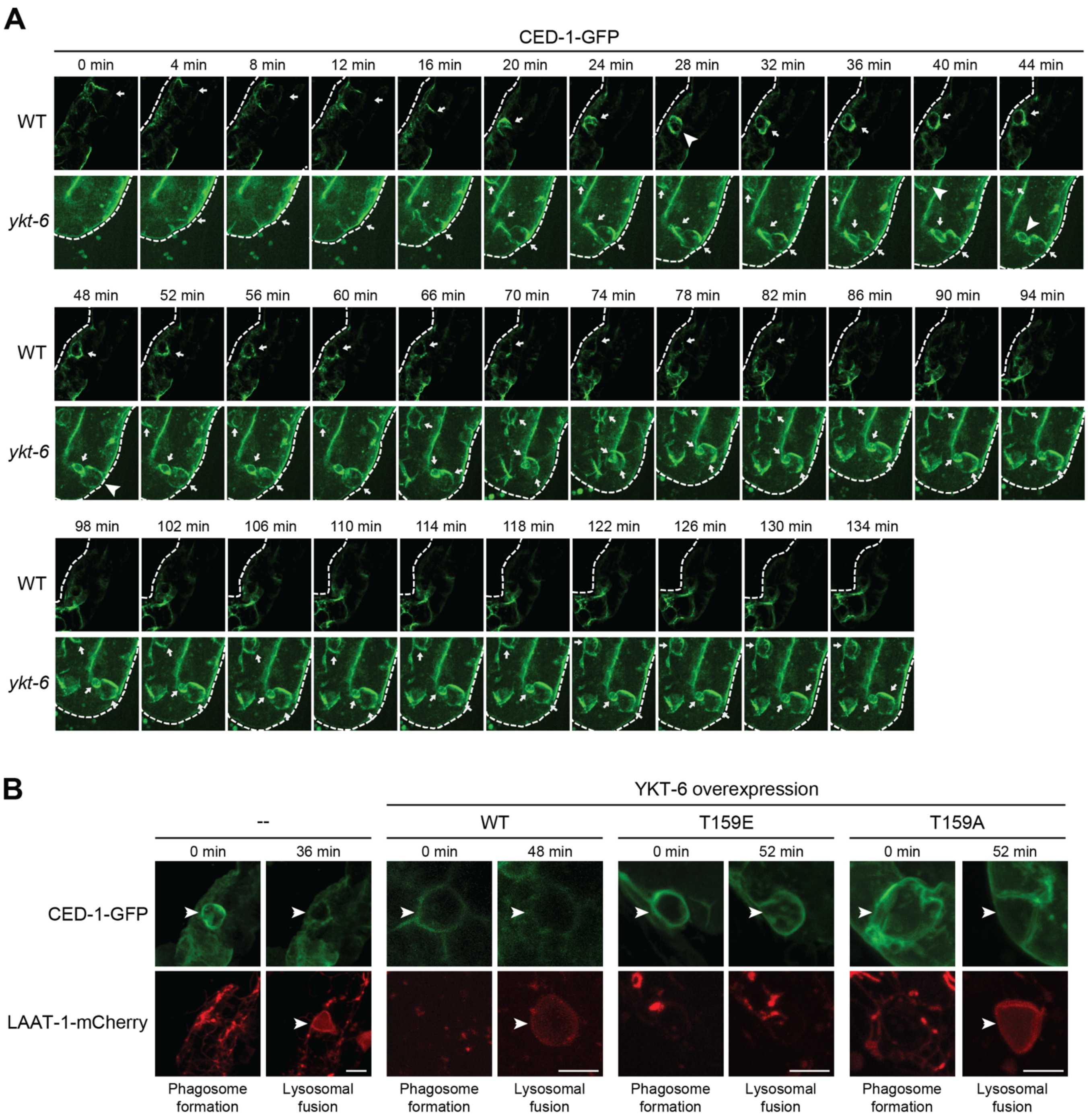

Fig. S4. YKT-6 phosphorylation inhibits phagosome-lysosome fusion

A) Time lapsed live cell images of control and ykt-6 RNAi treated worms from Fig 8B were acquired every 4 min to monitor the engulfment and degradation of cell corpses in the *C. elegans* germline. The engulfment of cell corpses by CED-1-GFP positive membranes is marked with a small arrow. The closure time is labeled with a large arrowhead. Images are representative of three independent biological replicates.

B) Time lapsed live cell images from Fig 8D. The indicated YKT-6 constructs were overexpressed selectively in sheath cells. The formation of phagosomes and their lysosomal degradation was monitored by visualization of CED-1-GFP and the lysosomal marker LAAT-1-mCherry. The arrowheads indicate the occurrence of phagosome closure and their fusion with lysosomes. Scale bar: 5 µm. Images are representative of three independent biological replicates.

Journal of Cell Science • Supplementary information

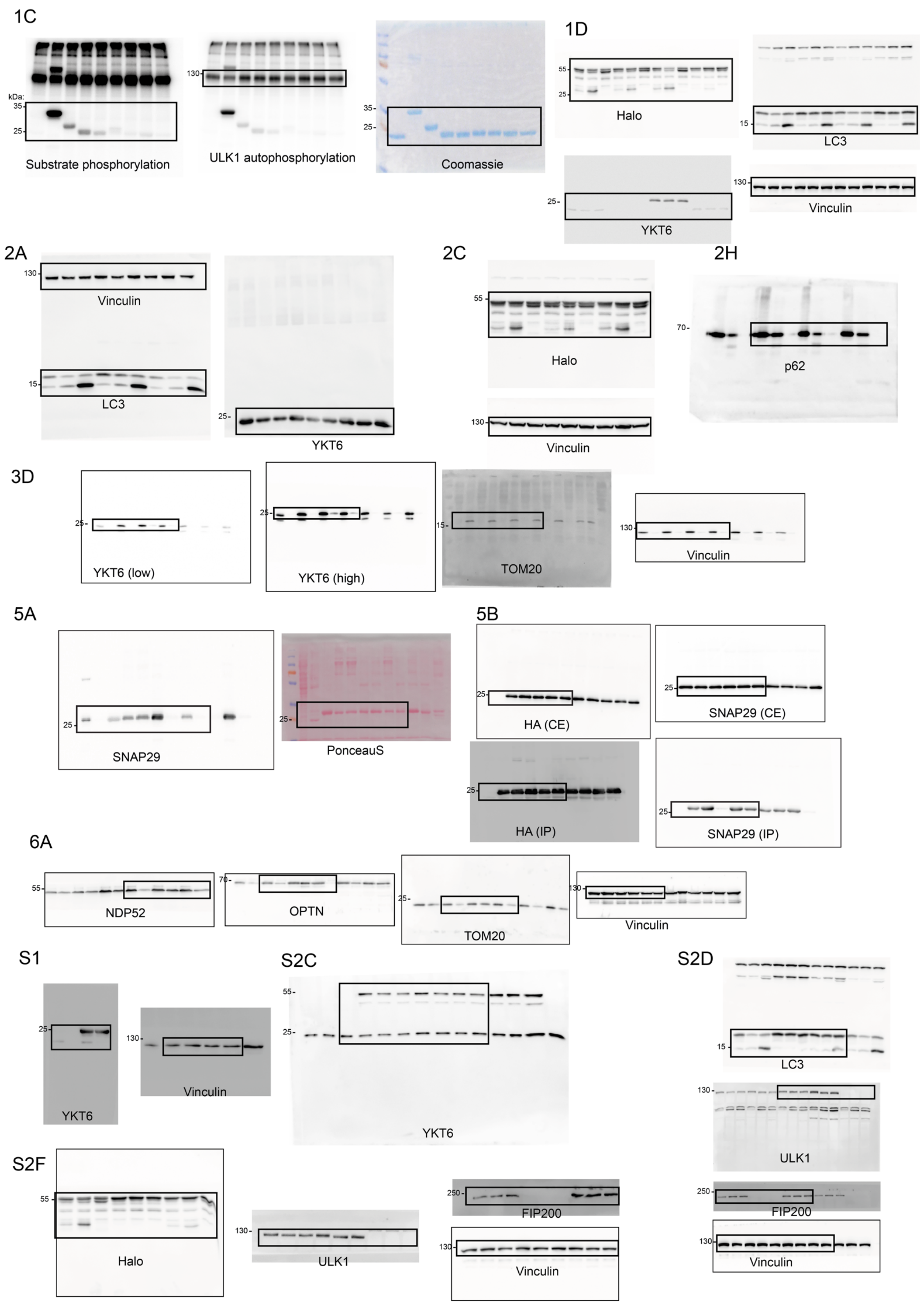

Fig. S5. Uncropped western blots

Journal of Cell Science • Supplementary information
