# Supplementary material for: ULK1-mediated phosphorylation regulates the conserved role of YKT6 in autophagy
Source: J Cell Sci. 2023 Feb 10;136(3):jcs260546. doi: 10.1242/jcs.260546 (PMC10022743; doi:10.1242/jcs.260546)
Supplement: Supplementary file 1 [file joces-136-260546_review_history.pdf]

ULK1-mediated phosphorylation regulates the conserved role of YKT6 in autophagy

Pablo Sánchez-Martín, Franziska Kriegenburg, Ludovico Alves, Julius Adam, Jana Elsaesser, Riccardo Babic, Hector Mancilla, Mariya Licheva, Georg Tascher, Christian Münch, Stefan Eimer and Claudine Kraft
DOI: 10.1242/jcs.260546

Editor: Tamotsu Yoshimori

Review timeline

Original submission:	19 August 2022
Editorial decision:	23 September 2022
First revision received:	7 December 2022
Accepted:	6 January 2023

Original submission

First decision letter

MS ID#: JOCES/2022/260546

MS TITLE: ULK1-mediated phosphorylation regulates the conserved role of YKT6 in autophagy

AUTHORS: Pablo Sanchez-Martin, Franziska Kriegenburg, Ludovico Alves, Jana Elsaesser, Riccardo Babic, Hector Mancilla, Mariya Licheva, Julius Adam, Georg Tascher, Christian Muench, Stefan Eimer, and Claudine Kraft
ARTICLE TYPE: Research Article

We have now reached a decision on the above manuscript.

To see the reviewers' reports and a copy of this decision letter, please go to: https://submit-jcs.biologists.org and click on the 'Manuscripts with Decisions' queue in the Author Area. (Corresponding author only has access to reviews.)

As you will see, the reviewers raise a number of substantial criticisms that prevent me from accepting the paper at this stage. They suggest, however, that a revised version might prove acceptable, if you can address their concerns. If you think that you can deal satisfactorily with the criticisms on revision, I would be pleased to see a revised manuscript. We would then return it to the reviewers.

Please ensure that you clearly highlight all changes made in the revised manuscript. Please avoid using 'Tracked changes' in Word files as these are lost in PDF conversion.

I should be grateful if you would also provide a point-by-point response detailing how you have dealt with the points raised by the reviewers in the 'Response to Reviewers' box. Please attend to all of the reviewers' comments. If you do not agree with any of their criticisms or suggestions please explain clearly why this is so.

Reviewer 1

Advance summary and potential significance to field

© 2023. Published by The Company of Biologists under the terms of the Creative Commons Attribution License (https://creativecommons.org/licenses/by/4.0/).

The manuscript entitled “ULK1-mediated phosphorylation regulates the conserved role of YKT6 in autophagy” by Pablo S.nchez-Martin et al. describes crucial role of phosphorylation on Ykt6 by Ulk1. It prevents premature bundling with the other SNARE proteins and suppresses autophagosome-lysosome fusion. Further, the authors demonstrated that knockdown of Ykt6 resulted in defect in autophagy also in nematode.

In general, data are clean and convincing. Although, main context are conserved in yeast several points are special in mammalian cells, and will be deserved to be reported in this journal. However, several concerns should be addressed before acceptance.

Comments for the author

1. Figure 2AB. The difference between WT and T156E are not so prominent and substantial autophagy still progressed, although quantitation of band intensity accompanies some difference. First, this blot should include Ykt6 knock down alone sample, and show the effect of T156E expression. Second, if Ykt6 knock down alone is not enough to suppress autophagy, additional knock down of Stx17 is required to show the effect of T156E expression in bulk autophagy.

2. If Ykt6 T156 phosphorylation prevent fusion of premature autophagosome, one possibility is that T156A expression leads to some related phenotypes. However, no phenotypes were observed in T156A expressed cells. What could be discussed?

<u>Reviewer 2</u>

Advance summary and potential significance to field

Previous studies including that from this authors' group revealed that the autophagy-related protein kinase Atg1 phosphorylates the SNARE protein Ykt6 to inhibit its bundle formation with other SNAREs and thereby prevents premature fusion of autophagosome intermediates with the vacuole in S. cerevisiae. In this study, the authors have extended their analysis to other organisms, mammalian cells and C. elegans. They showed that the mammalian Atg1 homolog UKL1 phosphorylates YKT6 at a threonine residue, T156, corresponding to one of those they reported to be phosphorylated by Atg1 in yeast. As with the case in yeast, the phospho-mimetic mutation T156E interfered with the YKT6 interaction with a cognate SNARE, SNAP29.

This mutation impaired both autophagosome formation and autophagosome-lysosome fusion, suggesting that the phosphorylation of T156 in YKT6 by ULK1 impedes these processes in autophagy. Additionally. the authors showed that YKT-6 is also important for autophagosome formation and autophagosome-lysosome fusion in C. elegans, although its phosphorylation by a ULK1 homolog has not been examined. In summary, this study for the first time describes the ULK1-mediated phospho-regulation of YKT6 in mammalian autophagy, but several issues remain to be addressed to strengthen the authors' conclusions or improve the manuscript.

Comments for the author

Specific comments:

(1) Although the T156E mutation impaired autophagosome formation in this study defects in autophagosome formation in YKT6-depleted cells were not described in previous studies. The authors should address this apparent discrepancy by examining autophagosome formation and autophagosome accumulation in YKT6 knockdown cells without rescue by the expression of wild-type YKT6 and add discussion based on the results.

(2) Fig. 1C: A control with a kinase-defective mutant of ULK1 is essential to conclude that ULK1 is responsible for YKT6 phosphorylation observed.

(3) Fig. 3A and B: The authors should confirm that LC3 puncta observed represent autophagosomes by simultaneously analyzing cells defective in autophagosome formation.

© 2023. Published by The Company of Biologists under the terms of the Creative Commons Attribution License (https://creativecommons.org/licenses/by/4.0/).

(4) Fig. 3E and F: This experiment to examine whether ULK1 is required for YKT6 association with autophagosomes seems not to make sense, because autophagosomes do not form in ULK1 KO cells.

(5) The most critical issue in the present manuscript is that the authors do not convincingly show defective autophagosome-lysosome fusion in the YKT6 mutant due to its defect in autophagosome formation (the results of the experiments shown in Fig. 4 can all be explained by defective autophagosome formation). I wonder if the authors could circumvent this difficulty by means of in vitro fusion assay. Alternatively, the authors may be able to count STX17-positive yellow tfLC3 puncta or ATG5-negative yellow tfLC3 puncta as complete autophagosomes.

(6) The authors propose that the phosphorylation of YKT6 by ULK1 acts to prevent premature autophagosome intermediate-lysosome fusion. However, this phosphorylation was also suggested to impede autophagosome formation. Then, when should ULK1 phosphorylate YKT6? The authors should discuss this point to clarify this discrepancy.

(7) Fig. 5A: To strengthen the conclusion that YKT6 phosphorylation by ULK1 inhibits the YKT6-SNAP29 interaction, the authors should do this experiment using wild-type YKT6 phosphorylated by ULK1 in vitro.

(8) Phagosome-lysosome fusion in C. elegans seems to be a good experimental system to investigate the fusogenic function of YKT-6 and its phospho-regulation separately from autophagosome formation. Given the advantage of this system, the authors should examine whether knockdown of a C. elegans ULK1 homolog affects phagosome-lysosome fusion to investigate whether YKT6 phospho-regulation by ULK1 homologs is also conserved in C. elegans.

(9) The authors should examine whether the components of the ULK1 complex, such as ATG13 and FIP200, are important for YKT6 phosphorylation.

First revision

Author response to reviewers' comments

Point by point reply to referee comments

We thank both referees for their insightful comments, which helped us to substantially improve our manuscript. As explained in detail below, we have addressed all points and added extensive new validation data, supporting our findings.

Reviewer 1 Advance Summary and Potential Significance to Field:

The manuscript entitled “ULK1-mediated phosphorylation regulates the conserved role of YKT6 in autophagy” by Pablo S.nchez-Martin et al. describes crucial role of phosphorylation on Ykt6 by Ulk1. It prevents premature bundling with the other SNARE proteins and suppresses autophagosome-lysosome fusion. Further, the authors demonstrated that knockdown of Ykt6 resulted in defect in autophagy also in nematode.

In general, data are clean and convincing. Although, main context are conserved in yeast, several points are special in mammalian cells, and will be deserved to be reported in this journal. However, several concerns should be addressed before acceptance.

Reviewer 1 Comments for the Author:

1. Figure 2AB. The difference between WT and T156E are not so prominent and substantial autophagy still progressed, although quantitation of band intensity accompanies some difference. First, this blot should include Ykt6 knock down alone sample, and show the effect of T156E expression. Second, if Ykt6 knock down alone is not enough to suppress autophagy, additional knock down of Stx17 is required to show the effect of T156E expression in bulk autophagy.

© 2023. Published by The Company of Biologists under the terms of the Creative Commons Attribution License (https://creativecommons.org/licenses/by/4.0/).

As suggested by the reviewer, we have included the phenotype of YKT6 RNAi. Depletion of YKT6 results in autophagy defects. We included different assays to monitor autophagy: LC3 lipidation, Halo-LC3 cleavage and LDH sequestration. These results are now shown in Figure 1D, E, F, G, S1B and C.

To substantiate our findings that the YKT6-T156E mutant results in an autophagy defect, we also performed additional autophagy assays with this mutant, the Halo-LC3 cleavage and LDH sequestration assay. As found for LC3 lipidation and puncta formation, the YKT6-T156E mutant showed an autophagy defect, further supporting our findings. These new results are now shown as Figure 2C, D, E.

2. If Ykt6 T156 phosphorylation prevent fusion of premature autophagosome, one possibility is that T156A expression leads to some related phenotypes. However, no phenotypes were observed in T156A expressed cells. What could be discussed?

One could expect an increase in autophagic activity for the T156A mutant due to enhanced formation and enhanced fusion, but also a defect in the T156A mutant could be expected, due to aberrant premature fusion, which would result in the release of the cargo into the cytosol when uncompleted autophagosomes fuse with lysosomes. These effects might compensate each other when monitoring autophagy flux. We see no significant difference between the T156A mutant and the WT in autophagy flux assays, however, we do see increased interaction of the T156A mutant with SNAP29. Therefore, there is a phenotype observed in the T156A mutant, which is enhanced bundling with SNARE proteins. We also performed co-immunoprecipitations of YKT6 from WT and ULK1-KO cells followed by mass spectrometry. We found that in the absence of ULK1, YKT6 shows enhanced SNAP29 binding, which further supports our findings.
We added the following text to the manuscript to point out this effect:

On the other hand, the T156A mutation in HA-YKT6 enhanced the association with SNAP29. Similar to the in vitro data, the SNAP29-YKT6 interaction was unaffected by the Ser181 mutation (Fig 5B). Mass-spectrometry confirmed these findings (Fig 5C) and furthermore showed a slightly increased binding of YKT6 to SNAP29 in ULK1 KO cells (Fig 5D and E), further supporting that ULK1 acts as a negative regulator of this interaction.

Reviewer 2 Advance Summary and Potential Significance to Field:

Previous studies including that from this authors' group revealed that the autophagy-related protein kinase Atg1 phosphorylates the SNARE protein Ykt6 to inhibit its bundle formation with other SNAREs and thereby prevents premature fusion of autophagosome intermediates with the vacuole in S. cerevisiae. In this study, the authors have extended their analysis to other organisms, mammalian cells and C. elegans. They showed that the mammalian Atg1 homolog UKL1 phosphorylates YKT6 at a threonine residue, T156, corresponding to one of those they reported to be phosphorylated by Atg1 in yeast. As with the case in yeast, the phospho-mimetic mutation T156E interfered with the YKT6 interaction with a cognate SNARE, SNAP29. This mutation impaired both autophagosome formation and autophagosome-lysosome fusion, suggesting that the phosphorylation of T156 in YKT6 by ULK1 impedes these processes in autophagy. Additionally. the authors showed that YKT-6 is also important for autophagosome formation and autophagosome-lysosome fusion in C. elegans, although its phosphorylation by a ULK1 homolog has not been examined. In summary, this study for the first time describes the ULK1-mediated phospho-regulation of YKT6 in mammalian autophagy, but several issues remain to be addressed to strengthen the authors' conclusions or improve the manuscript.

Reviewer 2 Comments for the Author:

Specific comments:
(1) Although the T156E mutation impaired autophagosome formation in this study, defects in autophagosome formation in YKT6-depleted cells were not described in previous studies. The authors should address this apparent discrepancy by examining autophagosome formation and autophagosome accumulation in YKT6 knockdown cells without rescue by the expression of wild-type YKT6 and add discussion based on the results.

© 2023. Published by The Company of Biologists under the terms of the Creative Commons Attribution License (https://creativecommons.org/licenses/by/4.0/).

Previous studies on mammalian YKT6 have only addressed the fusion step, but have not addressed if formation is also affected. We found that the T156E mutant not only inhibits fusion but also affects autophagosome formation, as fewer autophagosomes accumulate upon fusion inhibition by Bafilomycin A1 treatment (Fig. 2F and G). We have now also monitored the formation of LC3 puncta in ykt6-depleted cells compared to WT cells and found that LC3 puncta formation was decreased upon loss of YKT6 after Bafilomycin A1 treatment in comparison to WT, which indicates an autophagosome formation defect (Fig. 1F and G). Importantly, in Torin 1/EBSS treated cells, in which fusion can take place normally, cells depleted for *YKT6* showed an increase in the number of LC3 puncta compared to WT cells. This indicates that although autophagosome formation is decreased, formed autophagosomes fail to fuse with lysosomes, resulting in their accumulation.

We have furthermore performed an extensive analysis of both T156E mutant and siYKT6 treated cells: LC3 lipidation, Halo-LC3 cleavage and LDH sequestration. These results are now shown in Figure 1D, E, F, G, S1B and C for siYKT6 and Fig 2 A, B, C, D, E for the T156E mutant. LDH sequestration specifically monitors the formation of autophagosomes, as it measures the uptake of LDH into autophagosomes in Bafilomycin A1 treated cells, and clearly showed an impairment in YKT6 mutant or depleted cells, further supporting that not only autophagosome-lysosome fusion, but also autophagosome formation is regulated by YKT6.

We have included and discussed this more clearly in the revised manuscript.

(2) Fig. 1C: A control with a kinase-defective mutant of ULK1 is essential to conclude that ULK1 is responsible for YKT6 phosphorylation observed.

In vitro phosphorylation assays were done with recombinant ULK1 purchased from Sigma, unfortunately no kinase dead version is available. We have tried many approaches to express ULK1 ourselves. We have tried E.coli, Sf9 insect cells and yeast, by expression of ULK1 alone or together with mATG13 and FIP200. However, we were unable to obtain soluble protein.
We have also tried to in vitro phosphorylate mammalian YKT6 with yeast Atg1, as kinase dead Atg1 is available. However, yeast Atg1 does not phosphorylate the mammalian protein.

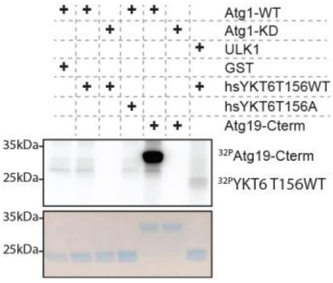

To nevertheless strengthen our findings, we performed co-immunoprecipitations of YKT6 from WT and ULK1-KO cells followed by mass spectrometry. We found that in the absence of ULK1, YKT6 shows enhanced SNAP29 binding, which further supports our findings. These results are shown in Figure 5D, E.

(3) Fig. 3A and B: The authors should confirm that LC3 puncta observed represent autophagosomes by simultaneously analyzing cells defective in autophagosome formation.

As suggested by the reviewer, we have analyzed LC3 puncta formation in control and in wortmannin treated WT cells. Upon autophagy inhibition by wortmannin treatment, LC3 puncta formation is largely lost. These results are shown in the new Figures S2 A and B.

(4) Fig. 3E and F: This experiment to examine whether ULK1 is required for YKT6 association with autophagosomes seems not to make sense, because autophagosomes do not form in ULK1 KO cells.

Mammalian ULK1 KO cells can still form LC3-positive structures and only show a partial defect in autophagy, which has previously been described (e.g. McAlpine et al., Autophagy 2013). We realize we did not make this point clear enough, and have included clarifications:

© 2023. Published by The Company of Biologists under the terms of the Creative Commons Attribution License (https://creativecommons.org/licenses/by/4.0/).

1. In Figure 1 D, S1B and C, and 3 E we include ULK1 KO cells in autophagy flux assays, showing that these are not completely defective (LC3 lipidation, Halo-LC3 cleavage, LDH sequestration). We also compare ULK1 KO, which show a partial defect, with fully defective FIP200 KO cells in Figure 3 E and S2 D-G.

2. We added a clarification to the text:
"ULK1 KO cells can still form autophagosomes though to a lesser degree than WT cells (McAlpine et al., 2013). Hence, autophagy in ULK1 KO cells is not completely defective compared to FIP200 KO cells (Fig 1D, Fig S1B and C, Fig 3E, Fig S2 D-G) (McAlpine et al., 2013). As no difference of YKT6 co-localization with GFP-LC3 in the presence and absence of ULK1 was observed (Fig 3F and G), we conclude that neither ULK1 nor phosphorylation at Thr156 alter the association of YKT6 with autophagosomal membranes."

(5) The most critical issue in the present manuscript is that the authors do not convincingly show defective autophagosome-lysosome fusion in the YKT6 mutant due to its defect in autophagosome formation (the results of the experiments shown in Fig. 4 can all be explained by defective autophagosome formation). I wonder if the authors could circumvent this difficulty by means of in vitro fusion assay.
Alternatively, the authors may be able to count STX17-positive yellow tfLC3 puncta or ATG5-negative yellow tfLC3 puncta as complete autophagosomes.

As suggested by the reviewer, we set up an *in vitro* fusion assay using separate fractions of lysosomes, autophagosomes and cytosol. Indeed, autophagosomes derived from YKT6-T156E mutant cells showed a defect in fusion, whereas autophagosomes from YKT6-T156A containing cells fused similar to such isolated from WT cells (Fig 4F, G). This nicely supports our findings.

We would like to point out that Fig 2 F also shows a strong indication for a fusion defect. If a mutant shows increased LC3 puncta, this can either mean that autophagosome formation is enhanced, or that fusion is defective. To distinguish between these two scenarios, cells are treated with the fusion inhibitor Bafilomycin A1. If autophagosome formation is enhanced, then Bafilomycin A1 treatment should further increase LC3 puncta. If fusion is defective, then Bafilomycin A1 should only give a minor increase in puncta. In our case the T156E mutant shows clear enhanced LC3 puncta formation in the absence of Bafilomycin A1, which only increase little upon Bafilomycin A1 treatment. This indicates that autophagosome-lysosome fusion is defective in these mutants. We tried to clarify this in the text.

Lastly, we replaced the quantification of the number of autolysosomes in Fig 4 B for the ratio between the number of autolysosomes and autophagosomes per cell, as this ratio better measures fusion defects, even if autophagosome formation is also affected. Using this ratio, as previously by quantifying the number of autolysosomes, we see a defective activity of the T156E mutant compared to the WT or T156A.

(6) The authors propose that the phosphorylation of YKT6 by ULK1 acts to prevent premature autophagosome intermediate-lysosome fusion. However, this phosphorylation was also suggested to impede autophagosome formation. Then, when should ULK1 phosphorylate YKT6? The authors should discuss this point to clarify this discrepancy.

Phosphomimicking on YKT6 clearly affects early and late steps in autophagy. We believe that ULK1-dependent phosphorylation of YKT6 is regulated in time and space. *In vivo*, ULK1 likely acts on YKT6 after YKT6 is recruited to autophagic membranes. As we find that ULK1 does not affect the recruitment of YKT6 to autophagic membranes, this could suggest that ULK1 phospho-regulation of YKT6 rather gets important later in the pathway at the autophagosome directly. The spatial regulation of YKT6 at the autophagosome would also prevent aberrant phospho-regulation of YKT6 molecules involved in other trafficking pathways. Further studies will be needed to clarify the precise timing of Ykt6 regulation.

We extended our discussion to emphasize this better.

© 2023. Published by The Company of Biologists under the terms of the Creative Commons Attribution License (https://creativecommons.org/licenses/by/4.0/).

"We therefore suggest that YKT6 function in different pathways is spatio-temporally regulated by different kinases, affecting either its membrane association and/or its function on membranes. In autophagy ULK1 is the main regulator of YKT6 acting on autophagosomal membranes"

(7) Fig. 5A: To strengthen the conclusion that YKT6 phosphorylation by ULK1 inhibits the YKT6-SNAP29 interaction, the authors should do this experiment using wild-type YKT6 phosphorylated by ULK1 in vitro.

We performed the requested experiment and see a reduction as expected (quantification of binding of 4 experiments). We do not observe a complete block in SNAP29 binding, likely due to substoichiometric phosphorylation *in vitro*, and/or because additional factors, which might be promoting ULK1 activity *in vivo*, are missing in the *in vitro* setup. As we described above, YKT6 phosphorylation is likely to take place on the autophagosome surface, after YKT6 lipid binding. It is then expectable that ULK1 is less efficient in phosphorylating soluble YKT6 *in vitro*.

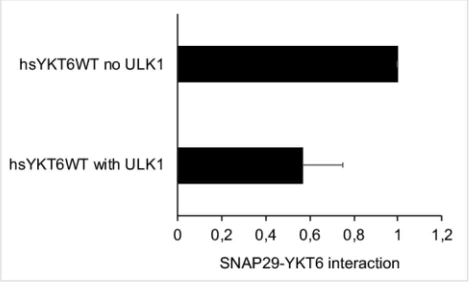

However, we also performed co-immunoprecipitations of YKT6 from WT and ULK1-KO cells followed by mass spectrometry, and found an enhanced binding of SNAP29 to YKT6 in KO cells, supporting our previous findings that ULK1 acts as a negative regulator of SNAP29-YKT6 association and thereby fusion. These results are now shown as new Fig 5 D,E.

(8) Phagosome-lysosome fusion in C. elegans seems to be a good experimental system to investigate the fusogenic function of YKT-6 and its phospho-regulation separately from autophagosome formation. Given the advantage of this system, the authors should examine whether knockdown of a C. elegans ULK1 homolog affects phagosome-lysosome fusion to investigate whether YKT6 phospho-regulation by ULK1 homologs is also conserved in C. elegans.

As ULK1 regulates autophagosome-lysosome fusion in a negative manner, the absence of ULK1 is expected to result in either no effect or enhanced fusion. We have performed the experiment as suggested, and found that unc-51 RNAi in sheath cells does not cause any strong phenotype.

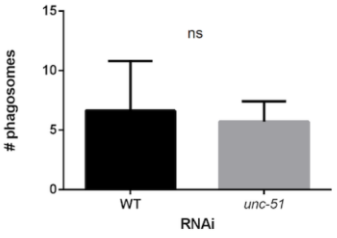

To test if *C.elegans* YKT-6 is also regulated by phosphorylation, we mutated the conserved threonine 159 to non- phosphorylatable alanine or phospho-mimetic glutamate. Expression of the *C.e.* YKT-6-T159E mutant in sheath cells resulted in a fusion defect while the *C.e.* YKT-6-T159A mutant sustained normal fusion similar to WT YKT6. These findings strongly support that the phospho-regulation found in yeast and mammals, is also conserved in worms. We added these results as new Fig 8D.

(9) The authors should examine whether the components of the ULK1 complex, such as ATG13 and FIP200, are important for YKT6 phosphorylation.

© 2023. Published by The Company of Biologists under the terms of the Creative Commons Attribution License (https://creativecommons.org/licenses/by/4.0/).

We agree with the reviewer that it would be interesting to address the role of ATG13 and FIP200 in YKT6 regulation in general, and that likely both ATG13 and FIP200 as part of the ULK1 complex are required for YKT6 phosphorylation in vivo. Detailed analysis of their role would require manipulation of ULK1 and/or the ULK1 complex. We have tried many approaches to express ULK1 ourselves. We have tried E.coli, Sf9 insect cells and yeast, both by expressing ULK1 alone or together with mATG13 and FIP200. However, we were unable to obtain soluble protein. As we speculate that YKT6 phosphorylation takes places on the surface of the autophagosome, in a physiological situation both ATG13 and FIP200 would be required for YKT6 phosphorylation by ULK1, as autophagosome formation cannot take place in the absence of either ATG13 or FIP200.

Second decision letter

MS ID#: JOCES/2022/260546

MS TITLE: ULK1-mediated phosphorylation regulates the conserved role of YKT6 in autophagy

AUTHORS: Pablo Sanchez-Martin, Franziska Kriegenburg, Ludovico Alves, Julius Adam, Jana Elsaesser, Riccardo Babic, Hector Mancilla, Mariya Licheva, Georg Tascher, Christian Muench, Stefan Eimer, and Claudine Kraft
ARTICLE TYPE: Research Article

I am happy to tell you that your manuscript has been accepted for publication in Journal of Cell Science, pending standard ethics checks.

Reviewer 1

Advance summary and potential significance to field

The authors responded appropriately to my concerns.

Comments for the author

The authors responded appropriately to my concerns.

Reviewer 2

Advance summary and potential significance to field

Although the authors could not address some of the issues I raised for the original manuscript experimentally due to technical difficulties, I find the revised manuscript is now suitable for publication in Journal of Cell Science.

Comments for the author

Although the authors could not address some of the issues I raised for the original manuscript experimentally due to technical difficulties, I find the revised manuscript is now suitable for publication in Journal of Cell Science.

© 2023. Published by The Company of Biologists under the terms of the Creative Commons Attribution License (https://creativecommons.org/licenses/by/4.0/).
